# Insights into the functional coordination of LigD and Ku in bacterial nonhomologous end joining

**DOI:** 10.1038/s41598-026-47294-z

**Published:** 2026-04-09

**Authors:** Alicia del Prado, Amalia Buitrago, Adrián de Rus-Moreno, Iza O. Bienkowska, Ana de Ory, Silvia Díaz-Arco, Miguel de Vega

**Affiliations:** Centro de Biología Molecular Severo Ochoa, Consejo Superior de Investigaciones Científicas-Universidad Autónoma de Madrid, Nicolás Cabrera 1, 28049 Madrid, Spain

**Keywords:** Ligase D, Ku, Nonhomologous end joining, DNA repair

## Abstract

Nonhomologous end joining (NHEJ) is a DNA repair pathway that directly ligates double-strand breaks (DSBs) and operates without a homologous template. In *Bacillus subtilis*, this process involves two core proteins: the Ku homodimer (Ku_*Bs*_) and LigD (LigD_*Bs*_). The ring-shaped Ku_*Bs*_ homodimer binds DNA ends, promotes their synapsis, and recruits LigD_*Bs*_. LigD_*Bs*_ is a multifunctional enzyme featuring an N-terminal ligase domain (LigDom) and a C-terminal polymerization domain (PolDom). PolDom, in addition to ribonucleotide insertion has an AP lyase activity that processes abasic sites (AP) near recessed 5′ ends. Here, it is studied whether a single LigD_*Bs*_ molecule can execute all three essential NHEJ catalytic steps, AP site incision, nucleotide insertion, and ligation in a coordinated manner when AP sites are present. The results indicate that LigD_*Bs*_ catalyzes these steps without dissociating from DNA, being this processive catalysis dependent on Ku_*Bs*_-mediated synapsis. We further show that the joining ability of Ku_*Bs*_ is critical for preventing intramolecular ligation of DNA ends, which otherwise would compromise DSB repair. Finally, the use of chimeric and truncated Ku variants revealed the specificity and dynamic nature of Ku_*Bs*_-LigD_*Bs*_ interactions during NHEJ, providing new mechanistic insights into bacterial DSB repair.

**Supplementary Information:**

The online version contains supplementary material available at 10.1038/s41598-026-47294-z.

## Introduction

Maintenance of genome integrity is essential for cell survival and genomic stability^1^. Double-strand breaks (DSBs) represent the most cytotoxic form of DNA damage, capable of causing chromosomal rearrangements, genetic information loss, or cell death if they are not properly repaired in a timely fashion^2^. Organisms have developed highly specialized mechanisms to repair this type of lesion, most notably homologous recombination (HR) and nonhomologous end joining (NHEJ)^3^. HR relies on the presence of a sister chromatid that provides the template for DNA synthesis through the break. This results in accurate repair of the lesion, being the main DSB repair pathway acting in rapidly proliferating bacteria and during the S and G2 phases of the eukaryotic cell cycle^2^. By contrast, NHEJ directly ligates the broken DNA ends without requiring a homologous template^4^. Although this repair pathway is active throughout the eukaryotic cell cycle, it is critical during the G1 phase, when the lack of a homologous template prevents DSB repair by homologous recombination^5^. In higher eukaryotes, NHEJ initiates with DNA ends recognition by the Ku70/Ku80 heterodimer. This complex threads onto the DSB ends, protecting them from cellular nucleases while serving as a hub to recruit the other components of the repair pathway, including DNA-PKcs, XRCC4-LigIV, XLF, PAXX and APLF, which promote and stabilize end synapsis^6^. While NHEJ can accurately repair DSBs, DNA ends are frequently incompatible for direct ligation requiring their prior processing by cellular nucleases (e.g., Artemis and SETMAR/Metnase) and/or DNA polymerases (e.g., Pol λ and Pol μ), frequently resulting in mutagenic outcomes^4,7,8^. In bacteria, NHEJ is predominantly active in organisms that spend much of their life cycle in the stationary phase, where only a single chromosome copy is available^9,10^. The *core* bacterial factors responsible for NHEJ are the Ku homodimer and the ATP-dependent Ligase D (LigD)^9,10^. Similar to higher eukaryotes, the ring-shaped Ku homodimer recognizes and threads onto the DSB ends, thereby protecting them from nucleolytic degradation^11,12^, promoting synapsis^13–16^, and recruiting LigD^16^. LigD is a multifunctional enzyme whose ligase domain (LigDom) is often fused to a polymerization domain (PolDom), that adds ribonucleotides to fill the potential gaps that result after the synapsis^9,10^, and to a phosphoesterase domain (PEDom) endowed with 3’-ribonuclease and 3’-phosphatase activities required for ribonucleotide resection of RNA intermediates to leave ligatable 3’-ends^17,18^. Variations exists regarding the presence and order of these domains within a single LigD protein^19,20^. Furthermore, many bacteria possessing the NHEJ repair system encode for several orthologues of Ku and/or of LigD domains^20^.

*Bacillus subtilis* is a Gram-positive spore-forming bacterium that possesses an NHEJ system comprising Ku (Ku_*Bs*_) and LigD (LigD_*Bs*_), with both genes being expressed during spore development^21^. This machinery protects stationary phase cells and spores against various DSB-inducing agents^21–24^. Ku_*Bs*_ binds and protects the ends^12^, promotes their bridging^16^, and recruits and interacts functionally with LigD_*Bs*_^12,25^. Ku_*Bs*_ consists of a central *core* domain through which DNA ends are threaded, and a C-terminal region that can be subdivided into two domains: a minimal C-terminal domain (minimal Cter) that interacts with LigD_*Bs*_, and an extended C-terminal domain (ext Cter) that limits Ku_*Bs*_ threading into DNA^12^. Although early findings suggested that the ext Cter domain was also involved in bridging the ends of a DSB^12^, it was later shown that a Ku_*Bs*_ variant lacking the entire C-terminal region could still maintain synapsis of the DNA ends^16^. Additionally, the same study provided evidence that the Ku_*Bs*_* core* domain also contributes to LigD_*Bs*_ interaction. Further research performed with *Mycobacterium tuberculosis* LigD (LigD_*Mt*_) and Ku (Ku_*Mt*_) highlighted the importance of the minimal Cter domain for LigD_*Mt*_ recruitment and ligation stimulation, as well as the critical role of the ext Cter domain not only for efficient DNA bridging but also for stimulating the LigD_*Mt*_ nick-sealing activity^26^. Similar to the *B. subtilis* system, Ku_*Mt*_* core* also interacts with LigD_*Mt*_. Therefore, altogether those studies reveal a division of labor among the different bacterial Ku regions. Beyond its role in bridging the ends of a break and in recruiting LigD_*Bs*_, Ku_*Bs*_ is also endowed with an AP/5’-dRP lyase activity that processes AP sites proximal to protruding 5’-ends^27^. After DSB repair is complete, Ku_*Bs*_ remains stably bound to the DNA, suggesting the need for an active mechanism to further remove it^16^. LigD_*Bs*_ has a bimodular conformation with an N-terminal LigDom and a C-terminal PolDom^25,28,29^. The latter, in addition to ribonucleotide insertion, has an AP-lyase activity that acts on AP-sites proximal to recessed 5’-ends during the NHEJ^30^. LigDom also possesses a 5’-dRP-lyase activity, which could remove a 5’-dRP group to enable subsequent gap filling and ligation by LigD_*Bs*_ during a base excision repair reaction, a fact that led to suggest that bacterial NHEJ proteins may participate in additional DNA repair pathways^31^.

Considering the multiple catalytic activities of LigD_*Bs*_, polymerization, ligation and AP processing, as well as the role of the different Ku domains mentioned above, the present work aims to study the ability of a single LigD_*Bs*_ molecule to sequentially and coordinately perform the three catalytic steps during NHEJ, as well as to ascertain the role of Ku_*Bs*_ regions in the functional and specific interaction with LigD_*Bs*_ during the NHEJ reaction. The results show that LigD_*Bs*_ can execute all the necessary catalytic steps for DSB repair without dissociating from the DNA. Furthermore, the data reveal dynamic changes in the Ku_*Bs*_-LigD_*Bs*_ interaction throughout the NHEJ process, highlighting their importance for proper execution of the process. Furthermore, the study shows that the Ku_*Bs*_-mediated synapsis of partially complementary ends is essential for preventing the intramolecular ligation of DNA ends by LigD_*Bs*_, an event that would otherwise compromise proper repair of the DSB.

## Materials and methods

### Proteins and reagents

Unlabeled nucleotides were purchased from Cytiva. Labeled nucleotide [γ^32^P]-ATP (3000 Ci/mmol) was from Revvity. Where indicated, ssDNA oligonucleotides were radiolabeled at the 5’ end with [γ^32^P]-ATP and T4 polynucleotide kinase (T4PNK). T4PNK and *Escherichia coli* uracil DNA glycosylase (UDG) were from New England Biolabs. Activated calf thymus DNA was from Sigma-Aldrich. LigD_*Bs*_ was purified as described^25^. The LigDom of LigD_*Bs*_ (residues 1–320) was purified as described^31^.

### DNA substrates

Oligonucleotides were from Integrated DNA Technologies. For clarity, sequences are shown in each figure, with the exception of upstream DNA hybrids BP1-6 that result from the hybridization of oligonucleotides BP1 (5’-CTCCACCGTACTGCGCATCAGCGGAACGAG), BP2 (5’-CTCCACCGTACTGCGCATCAGCGGAACGTG), BP3 (5’-CTCCACCGTACTGCGCATCAGCGG AACCTG), BP4 (5’-CTCCACCGTACTGCGCATCAGCGGAAGCTG), BP5 (5’-CTCCACCGTAC TGCGCATCAGCGGATGCTG) and BP6 (5’-CTCCACCGTACTGCGCATCAGCGGTTGCTG) to BP COMP (5’-PGCTGATGCGCAGTACGGTGGAG); and downstream DNA-C that results from the hybridization of oligonucleotides 5’-PAGCTGATGCGCAGTACGG-Cy5 and 5’-CCGTACTGCGCATCAGCTGCAGCAAG. The sequence of the length markers were: 45mer-Cy5 marker (5’-CCGTACTGCGCATCAGCTCGAGCAGAGAGCTGATGCGCAGTACGG-Cy5); Cy5-47mer marker (Cy5- CCGTTGGATCATATAGAGTACGCTGCTCGAGCTGATGCGCAGTACGG); and Cy5-47mer-Cy5 marker (Cy5-5’-CCGTTGGATCATATAGAGTACGCTGCTCGAGCTGATGCGCAGTACGG-Cy5. The dsDNA molecules were obtained by hybridizing the indicated oligonucleotides in the presence of 60 mM Tris–HCl (pH 7.5) and 0.2 M NaCl and heating to 90 °C for 5 min before slowly cooling to room temperature overnight.

### Obtention of Ku variants

Ku_*Bs*_, *Pseudomonas aeruginosa* Ku (Ku_*Pa*_)_*,*_ Ku_*BsPa*_ [(Ku_*Bs*_* core* (residues 1–236) + Ku_*Pa*_ C-terminal region (residues 239–293)], and Ku_*PaBs*_ [(Ku_*Pa*_* core* (residues 1–238) + Ku_*Bs*_ C-terminal region (residues 237–294)] were synthesized by Cusabio Technology and cloned between the restriction sites *Nde*I and *Bam*HI of the pET28a( +) vector to express the recombinant proteins fused to an N-terminal (His)_6_-tag (see schematics of the Ku variants in Fig. 6a). Ku_*Bs*_ protein has the same sequence as that described in^12^, which matches the annotated Ku protein from *B. subtilis* 168 (NP 389,224.1) from the initiator ATG at position 18. Truncated variants Ku_*Bs*_Δext (*core* + minimal Cter domains of Ku_*Bs*_) and Ku_*Pa*_Δext (*core* + minimal Cter domains of Ku_*Pa*_) were obtained by introducing stop codons at positions 260 and 266 of the genes coding for Ku_*Bs*_ and Ku_*Pa*_, respectively, using the Q5 site directed mutagenesis kit (New England Biolabs). The presence of the desired mutation and the absence of additional ones were determined by sequencing the entire gene. *Escherichia coli* BL21(DE3) cells were transformed with the corresponding recombinant expression plasmid. Transformed cells were grown overnight in LB medium at 37 ºC in the presence of 50 µg/mL kanamycin. Cells were diluted into the same media and incubated at 37 ºC until the DO_600_ reached 0.6. IPTG (Sigma) was then added to a final concentration of 1 mM. After additional 2 h at 30 ºC, cells were harvested and the pellet resuspended in Buffer A (50 mM Tris–HCl, pH7.5, 0.7 M NaCl, 7 mM β-mercaptoethanol, 5% glycerol, 10 mM imidazol) in the presence of protease inhibitors cocktail (Roche). Cells were disrupted by sonication and centrifuged for 20 min at 23 430 × g, to separate insoluble proteins from the soluble extract containing the corresponding recombinant Ku variant. The soluble fraction was loaded onto a Ni–NTA column (Qiagen) pre-equilibrated with Buffer A. The recombinant Ku proteins were eluted with Buffer A containing 50–100 mM imidazole (Ku_*Bs*_), 75–150 imidazole (Ku_*BsPa*_, Ku_*Bs*_Δext, Ku_*Pa*_), 50–200 mM imidazole (Ku_*PaBs*_) and 75–200 mM imidazole (Ku_*Pa*_Δext). Fractions containing the corresponding recombinant Ku variant were pooled and dialyzed againts Buffer B (50 mM Tris–HCl, pH7.5, 0.1 M NaCl, 7 mM β-mercaptoethanol, 5% glycerol) and further loaded onto a Heparin Sepharose column preequilibrated with the same buffer. The protein variants were eluted with Buffer B containing 0.3–0.35 M NaCl (Ku_*Bs*_), 0.4–0.6 M NaCl (Ku_*BsPa*_ and Ku_*PaBs*_), 0.3–0.4 M NaCl (Ku_*Bs*_Δext), 0.4–0.5 M NaCl (Ku_*Pa*_), and 0.3–0.5 M NaCl (Ku_*Pa*_Δext). Finally, the purified variants were dialyzed against a buffer B containing 0.3 M NaCl, 50% glycerol and 1 mM EDTA, and stored at -20 ºC. Final purity of the protein was estimated to be > 90% by SDS-PAGE followed by Coomassie blue staining (see Supplementary Fig. S1).

### End joining of partially complementary 3’-protruding DNA ends in the presence of challenging DNA

The reaction mixture contained, in a final volume of 12.5 µL, 30 mM Hepes (pH 7.5), 4% (v/v) glycerol, 20 mM NaCl, 0.01% Tween-20, 1 mM DTT, 20 nM DNA-A, 25 nM DNA-Y, 100 nM Ku_*Bs*_/LigD_*Bs*_ complex (formed by preincubating both proteins 15’ at 4 ºC), and 1 µM CTP. The mixture was incubated for 5 min at 4 °C to allow the formation of the (Ku_*Bs*_/LigD_*Bs*_)-DNA complex. The reaction was initiated by the simultaneous addition of 0.6 mM MnCl_2_ and the indicated concentration of calf-thymus DNA as a trap. As a control for the efficiency of the trap, parallel reactions were performed in which the challenging DNA was mixed with substrates DNA-A and DNA-Y before adding 100 nM Ku_*Bs*_/LigD_*Bs*_ complex. In these cases, reactions were initiated by adding 0.6 mM MnCl_2_. After incubation for 30 min at 30 °C the reactions were stopped by adding EDTA to 10 mM. Reactions products were resolved by 7 M urea-20% PAGE and visualized using a Typhoon scanner (Cytiva).

### End-joining of partially complementary DNA ends with near terminal AP sites

The reaction mixture contained, in a final volume of 12.5 µL, 30 mM Hepes (pH 7.5), 4% (v/v) glycerol, 20 mM NaCl, 0.01% Tween-20, 1 mM DTT, 100 nM CTP, 20 nM DNA-A and 40 nM of either DNA-B or DNA-Y both harboring a 2’-deoxyuridine site proximal to the 5’-recessive end. When indicated, natural AP sites were generated by further incubation with 0.2 U of *E. coli* UDG at 37 °C for 15 min. Subsequently, 50 nM Ku_*Bs*_/LigD_*Bs*_ complex was added. Reactions were started by adding 0.6 mM MnCl_2_. After incubation for 30 min at 30 °C reactions were stopped by adding EDTA to 10 mM. When indicated, the presence of AP sites was tested by adding 50 mM NaOH and further incubation for 5 min at 95 °C. Reactions products were resolved by 7 M urea-20% PAGE and visualized using a Typhoon scanner (Cytiva).

### End-joining of partially complementary DNA ends with near terminal AP sites in the presence of challenging DNA

The assay was performed essentially as described in the above section. In this case the reaction was initiated by the simultaneous addition of 0.6 mM MnCl_2_ and the indicated concentration of calf-thymus DNA as a trap. As a control of the efficiency of the trap, parallel reactions were performed in which the challenging DNA was mixed with substrates DNA-A and DNA-B before adding 100 nM Ku_*Bs*_/LigD_*Bs*_ complex. In these cases, reactions were initiated by adding 0.6 mM MnCl_2_. After incubation for 30 min at 30 °C the reactions were stopped by adding EDTA to 10 mM. When indicated, the presence of AP sites was tested by adding 50 mM NaOH and further incubation for 5 min at 95 °C. Reactions products were resolved by 7 M urea-20% PAGE and visualized using a using a Typhoon scanner (Cytiva).

### 3’-protruding end sequence requirements for the formation of inter/intramolecular ligation products.

The reaction mixture contained, in a final volume of 12.5 µL, 30 mM Hepes (pH 7.5), 4% (v/v) glycerol, 20 mM NaCl, 0.01% Tween-20, 1 mM DTT, 100 nM of the indicated ribonucleotide, and 20 nM of the specified DNA hybrid (see schematics in Fig. 3). In the case of the 2’-deoxyuridine-containing DNA substrates, they were previously treated with *E. coli* UDG (0.2 U) for 15 min at 37 °C to get natural AP sites. Subsequently, mixtures were supplemented with 25 nM LigD_*Bs*_ and 100 nM of the indicated ribonucleotide. The reaction was started by adding 0.6 mM MnCl_2_. After incubation for 10 min at 30 °C reactions were stopped by adding EDTA to 10 mM. When indicated, the presence of AP sites was tested by adding 50 mM NaOH and further incubation for 5 min at 95 °C. Reactions products were resolved by 7 M urea-20% PAGE and visualized using a using a Typhoon scanner (Cytiva).

### Discrimination between end-joining and folding-back conformations

The assay was carried out essentially as described in previous sections in the presence of 4 nM of the indicated non-labeled DNA substrate (see sequences in Fig. 4), 100 nM of the specified ribonucleotide and 100 nM LigD_*Bs*_. After incubation for 15 min at 30 °C reactions were stopped by adding EDTA up to 10 mM. Reactions products were resolved by 7 M urea-20% PAGE and visualized using a using a Typhoon scanner (Cytiva).

### Determination of the number of 3’-overhanging nucleotides to form a hairpin loop

The assay was performed as described above, in the presence of 5 nM of the indicated DNA with 100 nM LigD_*Bs*_ and 100 nM UTP. After incubation for 8 min at 30 °C, the reactions were stopped by adding EDTA up to 10 mM. Reactions products were resolved by 7 M urea-20% PAGE and visualized using a using a Typhoon scanner (Cytiva).

### Determination of the minimal base complementarity between protruding 3’-ends to allow end-joining

The assay was performed as described above, incubating 20 nM of labeled downstream DNA with either 20 nM or 40 nM of the indicated non-labeled BP hybrid (see Fig. 5), 100 nM LigD_*Bs*_, 100 nM Ku_*Bs*_ (where indicated), 1 µM CTP and 0.6 mM MnCl_*2*_. After incubation for 30 min at 37 °C, the reactions were stopped by adding EDTA up to 10 mM. Reactions products were resolved by 7 M urea-20% PAGE and visualized using a using a Typhoon scanner (Cytiva).

### Effect of the Ku variants in the NHEJ reaction

The incubation mixture (12.5 µL) contained 50 mM Tris–HCl pH 7.5, 0.6 mM MnCl_2_, 1 mM DTT, 4% (v/v) glycerol, 0.1 mg/mL BSA, 20 mM NaCl, 0.01% Tween-20, 100 nM CTP (in those assays where insertion + ligation activities are tested), 20 nM of the indicated substrates (Fig. 6), 50 nM of the indicated Ku variant and either 100 nM LigD_*Bs*_, 20 nM LigDom or 50 nM LigD_*Pa*_, as specified. After incubation at 37 °C for the indicated times, the reactions were stopped by adding EDTA up to 10 mM. Reactions products were resolved by 7 M urea-20% PAGE and visualized using a using a Typhoon scanner (Cytiva).

## Results

### A single LigD_***Bs***_ molecule is capable of performing the multiple enzymatic activities required during the NHEJ reaction

**Fig. 1:**
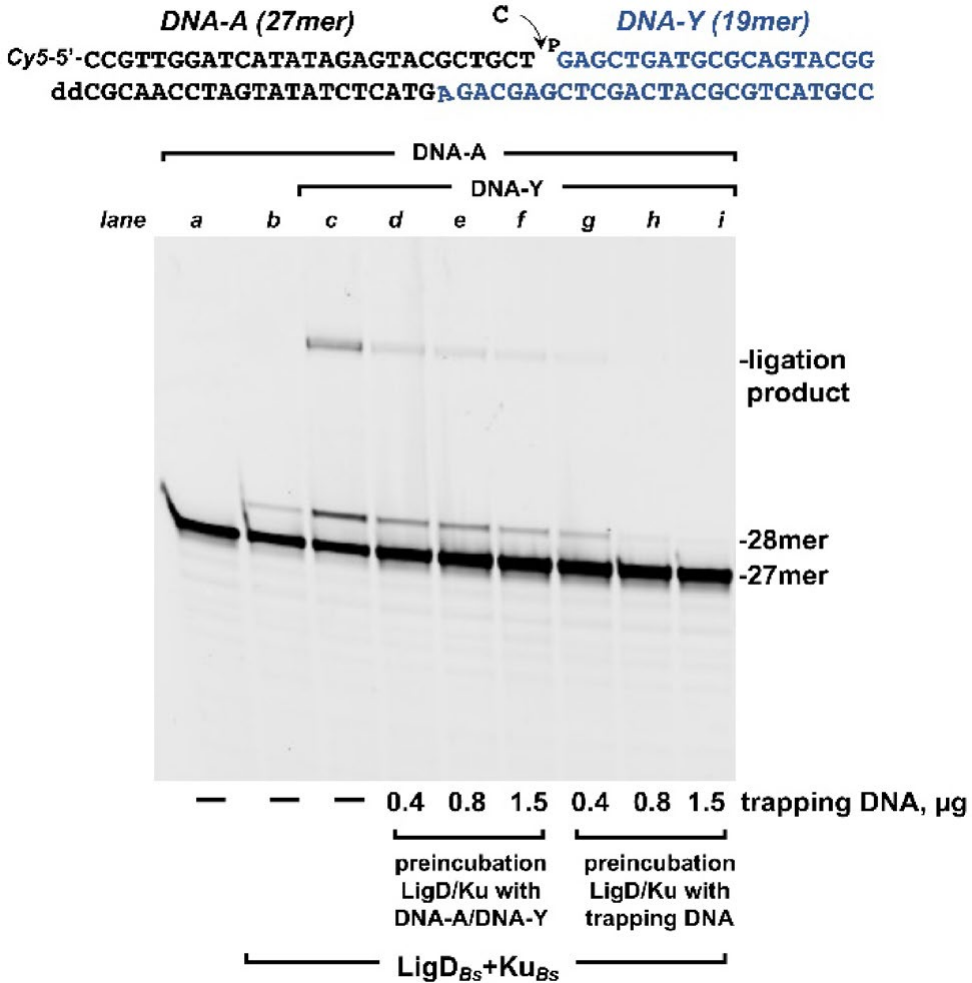
*NHEJ under single DNA binding conditions*. The assay was carried out as described in Materials and Methods by incubating 100 nM LigD_*Bs*_/Ku_*Bs*_ complex with 1 µM CTP, 20 nM DNA-A, and 25 nM DNA-Y, either in the absence (lanes *b-f*) or presence (lanes *g-i*) of trapping DNA. Reactions were initiated by adding either the metal activator alone (lanes *b, g-i*) or the metal activator together with the indicated amount of trapping DNA (lanes *d-f*).

As described above, LigDs possess all the catalytic activities to sequentially fill the potential gaps that result after the synapsis of DNA ends of a break and to ligate them to finish the repair reaction^9,10^. This fact prompted us to investigate whether a single LigD_*Bs*_ molecule carries out all reactions without dissociating from the DNA substrate. To test this, we designed an end-joining assay using calf-thymus activated DNA as a trap intended to capture LigD_*Bs*_ molecules that might dissociate during any step of the end-joining reaction, thereby preventing their re-association with the substrate (a methodology previously described for measuring, among others, the processivity of DNA polymerases^32,33^). Specifically, the LigD_*Bs*_/Ku_*Bs*_ complex was incubated with the two 3’ protruding DNA hybrids depicted in Fig. 1 (only DNA-A was 5’ labeled with Cy5). Upon synapsis, these substrates create a 1-nucleotide gap that could be filled due to the presence of CTP and Mn^2+^ ions in the reaction mixture. As shown in Fig. 1 (lane *c*), in the absence of trapping DNA, the synapsis between the DNA molecules permitted LigD_*Bs*_ to successfully accomplish the template-directed insertion of CMP at the 3’-end of the labeled strand (28mer band), followed by sealing of the nick to produce a fully repaired product (51% ± 1%, Mean ± SD ligation products respect to + 1 elongated DNA-A). A similar result was obtained when the reaction was initiated by the simultaneous addition of Mn^2+^ and increasing amounts of trapping DNA (Fig. 1, lanes *d*-*f*). The reduction in the amount of products indicates that only a small fraction of LigD_*Bs*_ molecules were stably bound to the DNA substrates before the reaction was started. The absence of 5’-labeled elongation and ligation products when the LigD_*Bs*_/Ku_*Bs*_ complex was preincubated with 0.8–1.5 µg of trapping DNA (Fig. 1, lanes *h* and *i*) reflects the effectiveness of the trap. This result allows us to conclude that both nucleotide insertion and subsequent ligation were carried out by the same LigD_*Bs*_ molecule without dissociation from the DNA substrate. The extension product observed in the absence of DNA-Y in lane *b* would probably correspond to a terminal transferase activity of LigD_*Bs*_, as described in other bacterial LigDs^9,10^.

Our previous results demonstrated that the NHEJ proteins Ku_*Bs*_ and LigD_*Bs*_ possess an AP lyase activity that enables them to process AP sites in a specific fashion^27,30,31^. Thus, whereas Ku_*Bs*_ preferentially removes AP sites at protruding 5’ ends, LigD_*Bs*_ incises AP sites proximal to recessed 5’-termini. In the latter case, incision on the AP site requires the binding of both, Mn^2+^ ions and the ribonucleotide complementary to the orphan base (the nucleotide placed opposite the AP site) at the catalytic site of the PolDom^30^. Once the AP site is incised, the resulting 5’P-end is stably bound by the PolDom, leading to the formation of the preternary-precatalytic complex. This complex is then poised to catalyze the further in *trans* addition of the ribonucleotide to the 3’-end of an incoming primer. The resulting nick is subsequently sealed by the ATP-dependent DNA ligase activity of the enzyme, completing the repair process^30^.

**Fig. 2:**
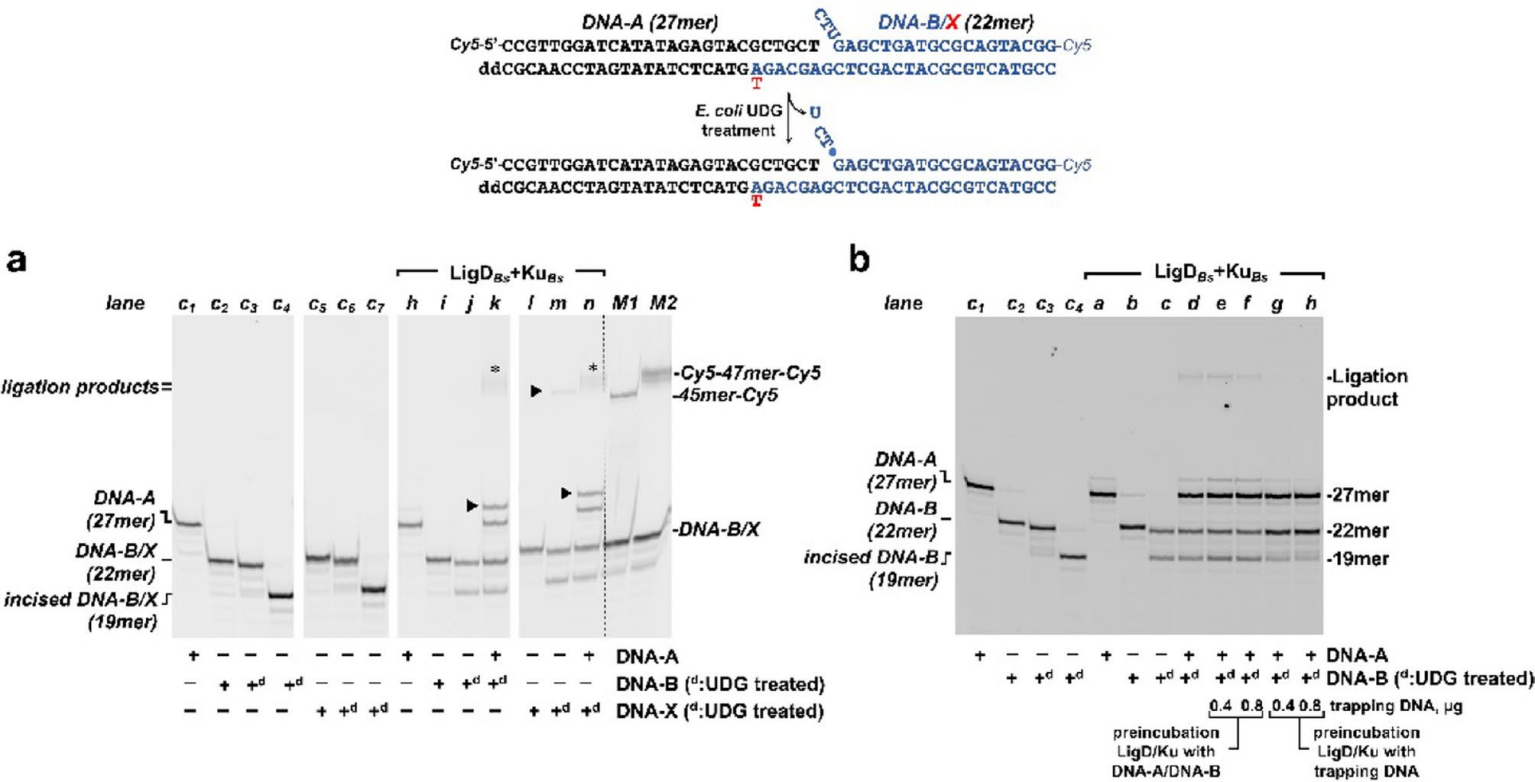
*Analysis of NHEJ by LigD*_*Bs*_*/Ku*_*Bs*_*.* (**a**) NHEJ of partially complementary DNA ends containing a terminal AP site. The assay was carried out as described in Materials and Methods, incubating 20 nM DNA-A and 40 nM DNA-B/X (depicted at the top of the figure) with 50 nM LigD_*Bs*_/Ku_*Bs*_ complex, and with 100 nM CTP. Where indicated, substrates DNA-B and DNA-Y were pretreated with UDG (+ ^d^) to generate a natural AP site. The figure is a composite image made from different parts of the same experiment (see Supplementary Information). M1: markers DNA-B + 45mer-Cy5; M2: markers DNA-X + Cy5-47mer-Cy5. C_1_: control DNA-A incubated without LigD_*Bs*_/Ku_*Bs*_; C_2_: control DNA-B incubated without LigD_*Bs*_/Ku_*Bs*_; C_3_: UDG-treated DNA-B incubated without LigD_*Bs*_/Ku_*Bs*_; C_4_: alkaline hydrolysis of the UDG-treated DNA-B; C_5_: control DNA-X without LigD_*Bs*_/Ku_*Bs*_, C_6_: UDG-treated DNA-X incubated without LigD_Bs_/Ku_*Bs*_; C_7_: alkaline hydrolysis of the UDG-treated DNA-X. (**b**) NHEJ of partially complementary DNA ends containing a terminal AP site under single DNA binding conditions. The assay was carried out as described in Materials and Methods by incubating 50 nM LigD_*Bs*_/Ku_*Bs*_ complex with 100 nM CTP and 20 nM of the specified NHEJ substrate (DNA-A, DNA-B), either in the absence (lanes C_1_-*f*) or presence (lanes *g*, *h*) of trapping DNA. Where indicated, DNA-B was pretreated with UDG to generate a natural AP site (+ ^d^). Reactions were initiated by adding either the metal activator alone (lanes C_1_-*d*, *g* and *h*) or the metal activator together with the indicated amount of trapping DNA (lanes *e*, *f*). C_1_: control DNA-A incubated without LigD_*Bs*_/Ku_*Bs*_; C_2_: control DNA-B incubated without LigD_*Bs*_/Ku_*Bs*_; C_3_: UDG-treated DNA-B incubated without LigD_*Bs*_/Ku_*Bs*_; C_4_: alkaline hydrolysis of the UDG-treated DNA-B.

To determine whether a single LigD_*Bs*_ molecule can perform all three catalytic reactions, incision on the AP site, in *trans* nucleotide addition, and ligation, a control end-joining reaction was first conducted using the substrates depicted in the upper part of Fig. 2. The upstream 5’-labeled DNA-A was incubated with the 3’-labeled downstream substrates DNA-B or DNA-X. These downstream substrates contain an internal 2’-deoxyuridine and differ only in the 3’-terminal nucleotide of the non-labeled strand, A or T, respectively (see schematics in Fig. 2). Incubation of the LigD_*Bs*_/Ku_*Bs*_ complex with the individual DNA substrates (DNA-A, DNA-B and DNA-X) in the presence of CTP and Mn^2+^ did not yield any reaction product (Fig. 2a, lanes *h*, *i* and *l*, respectively). AP sites (represented by a full circle in the schematics of the substrates) were generated by treating the DNA substrates DNA-B and DNA-X with *Escherichia coli* uracil DNA glycosylase (UDG), and their presence was confirmed by alkaline hydrolysis (the 19mer product in Fig. 2a, lanes *c*_*4*_ and *c*_*7*_). As expected, LigD_*Bs*_ incised the AP site on the UDG-pretreated downstream substrates DNA-B (Fig. 2a, lane* j*) and DNA-X (Fig. 2a, lane *m*),. The simultaneous presence of DNA-A and either DNA-B or DNA-X allowed LigD_*Bs*_ to insert CMP at the 3’-end of the upstream DNA-A substrate (indicated by an arrowhead in lanes *k* and *n* of Fig. 2a) and to seal the resulting nick, yielding a fully-repaired Cy5-47mer-Cy5 product [indicated by an asterisk in lanes *k* (46% ± 4%, Mean ± SD ligation products respect to 19mer incised products) and *n* (45% ± 2%, Mean ± SD ligation products respect to 19mer incised products) of Fig. 2a]. The reduction in the amount of ligation products generated by LigD with these substrates, compared with those obtained when prior AP incision is not required (Fig. 1), is likely due to the incomplete cleavage of the AP site by the AP lyase activity of LigD (compare the amount of 19mer product generated by LigD with that produced by the alkaline treatment). Notably, incubation of LigD_*Bs*_/Ku_*Bs*_ with the DNA-X substrate which harbors a terminal dTMP at the protruding 3’-end, resulted in a product with an electrophoretic mobility slightly faster than the Cy5-47mer-Cy5 product marker that represents the 27% ± 5% (Mean ± SD) of DNA-X, (indicated by an arrowhead in lane *m* of Fig. 2a, see below). In a similar assay started by adding the metal activator and 0.4–0.8 µg of trapping DNA (Fig. 2b), the LigD_*Bs*_/Ku_*Bs*_ complex generated all three expected products: the AP lyase product (19mer), the CMP insertion product (28mer) and ligation product (47mer) (Fig. 2b, lanes *e, f*). This result indicates that even on these substrates, the same LigD_*Bs*_ molecule catalyzes all three reactions [note the absence of reaction products when the proteins were preincubated with the trapping DNA (Fig. 2b, lanes *g* and *h*), demonstrating the effectiveness of the trap].

### LigD_***Bs***_ performs intramolecular ligation of DNA ends

**Fig. 3:**
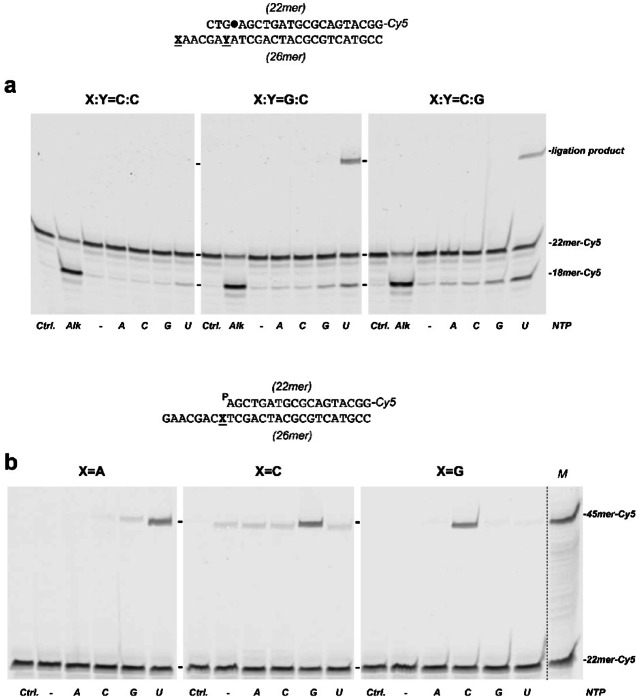
*Analysis of the nucleotide sequence requirements for self-ligation of the downstream DNA substrate.* Analysis of the NHEJ by LigD_*Bs*_/Ku_*Bs*_. (**a**) Effect of the complementarity between the terminal base of the protruding 3’-end and the base preceding the orphan nucleotide in the formation of the ligation product. A schematic of the DNA substrates is presented at the top of the figure. The 3’-terminal nucleotide (X) and the one preceding the orphan base (Y) are underlined. The filled circle in the sequence indicates the AP site resulting after treatment with *E. coli* UDG. The assay was performed as described in Materials and Methods by incubating 25 nM LigD_*Bs*_ with 20 nM of the indicated substrate in the presence of 100 nM of the specified ribonucleotide. *Ctrl*. lanes correspond to a control of the initial DNA before starting the reaction and incubated in the absence of protein. *Alk*, alkaline hydrolysis of the AP site. The figure is a composite image made from different parts of the same experiment (see Supplementary Information). (**b**) Analysis of the complementarity between the incoming nucleotide and the base adjacent to the 5’P-end. A schematic of the DNA substrates is presented at the top of the figure. The nucleotide adjacent to the 5’P-end is underlined. The assay was performed as described in Materials and Methods by incubating 25 nM LigD_*Bs*_ with 20 nM of the indicated DNA hybrid in the presence of 10 nM of the specified ribonucleotide. After incubation for 10 min at 30 °C, the reactions were stopped by adding EDTA to 10 mM. *Ctrl*. lanes correspond to a control of the initial DNA before starting the reaction and incubated in the absence of protein. The figure is a composite image made from different parts of the same experiment.

As mentioned above, the incubation of LigD_*Bs*_/Ku_*Bs*_ with DNA-X yielded a product shorter than the Cy5-47mer-Cy5 product, suggesting that a single nucleotide difference at the 3’-end of the protruding strand facilitates an unusual self-ligation of the downstream DNA molecule. In the case of DNA-X, the 3’-terminal dTMP of the substrate is complementary to the dAMP located immediately upstream of the orphan nucleotide base (dGMP). Based on this observation, we hypothesized that the formation of this self-ligation product relies solely on the formation of a dT:dA base pair preceding the base pair formed between the incoming CTP and the orphan dGMP. To test this, three new dsDNA substrates were designed, each containing an internal 2’-deoxyuridine at the fourth position from the recessed 5’-end. These substrates differed either in the 3’-terminal base of the protruding strand (dCMP in the left and right panels of Fig. 3a; dGMP in the middle panel) or in the base preceding the orphan nucleotide (dCMP in the left and middle panels of Fig. 3a; dGMP in the right panel). After treatment with UDG to generate the AP sites, the substrates were incubated with LigD_*Bs*_ and the indicated ribonucleotide. As expected, maximum AP lyase activity was observed in the presence of UTP, which is complementary to the orphan nucleotide^30^. As demonstrated in Fig. 3a, when the 3’-terminal nucleotide of the protruding strand was not complementary to the base immediately preceding the orphan nucleotide (left panel), LigD_*Bs*_ produced only the 18mer degradation product resulting from the AP site incision. By contrast, ligation products were clearly observed with substrates that allowed base pairing at this position (middle and right panels).

To determine whether the AP site processing favors self-ligation of the DNA, we assayed DNA substrates that mimic the products generated after AP site incision. These substrates contained a perfectly paired recessed 5’-P terminus and differed only in the nucleotide adjacent to this end (underlined in Fig. 3b). In all designed substrates the terminal nucleotide of the protruding 3’-end (dGMP) and the nucleotide preceding the one adjacent to the 5’P-end (dCMP) were complementary, allowing them to form a base pair. Incubation of these DNA substrates with LigD_*Bs*_ yielded 45mer ligation products. Product formation was observed, primarily in the presence of the ribonucleotide complementary to the base nearest the 5’-P end. This result leads to two key conclusions: self-ligation does not require a preceding AP lyase activity on an AP site; and, once LigD_*Bs*_ positions the template nucleotide in its polymerase active site, it can add the complementary ribonucleotide to the 3’-protruding end, coupling this addition directly to ligation with the 5’-P terminus. A 45mer ligation product was also detected even in the presence of a non-complementary nucleotide (e.g., GTP in the left panel of Fig. 3b), likely attributable to the low insertion fidelity of LigD_*Bs*_^25^. In the case of the substrate with dCMP serving as the templating nucleotide (middle panel), a shorter 44mer ligation product was obtained even in the absence of any added ribonucleotides. This suggests direct ligation between the 5’-P and the 3’-protruding end, facilitated by base complementarity between the 3’-terminal dGMP and the dCMP adjacent to the 5’P.

**Fig. 4:**
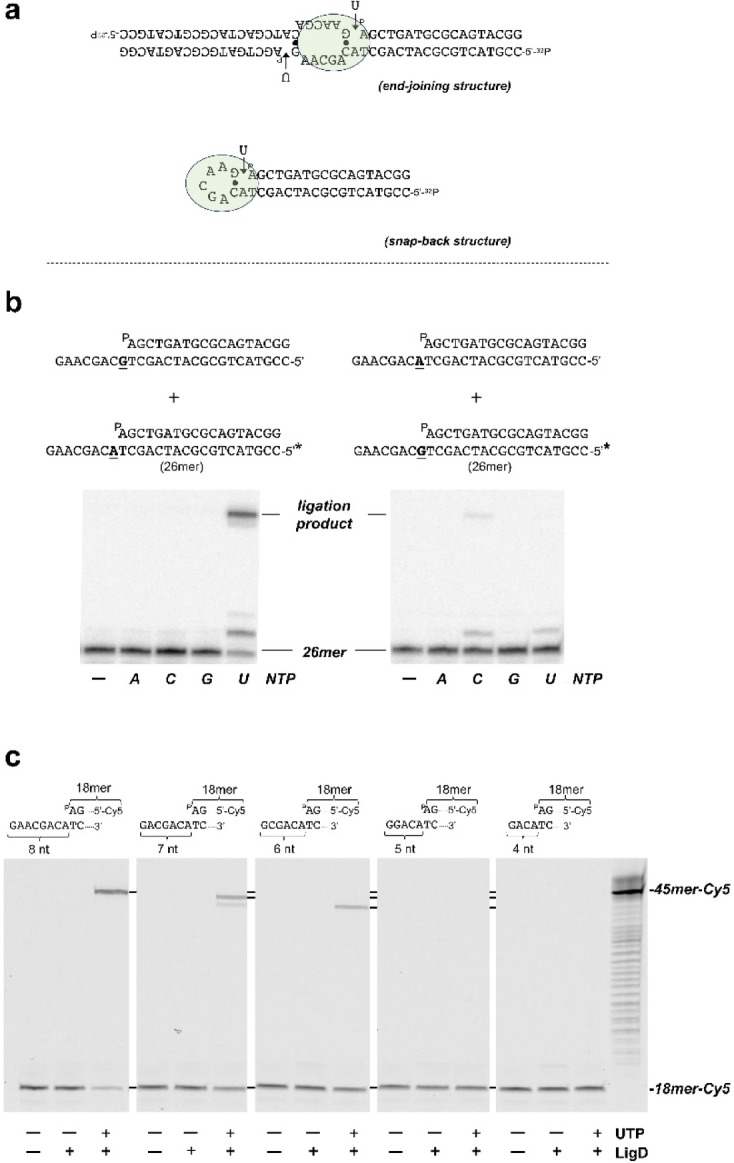
*Template-directed extension of the protruding 3’-end by LigD*_*Bs*_. (**a**) Schematic representation of the two structures that can enable template-directed nucleotide insertion at the protruding 3’ end. At the top, two DNA molecules could form an end-joining structure in which LigD_*Bs*_ (represented as a green oval) would carry out the in *trans* addition (intermolecular) of the nucleotide. At the bottom, a description of how the protruding 3’ end could form a snap-back-like structure through intramolecular base pairing between the terminal 3’ nucleotide and the base adjacent to the 5’P end, followed by elongation by LigD_*Bs*_. (**b**) Discrimination between the intra- and intermolecular models for 3’ extension by LigD_Bs_*.* The assay was carried out as described in Materials and Methods by incubating 4 nM of the non-labeled DNA and 1 nM of the 5’-labeled substrate represented in the upper part of the figure (the substrates differ in the underlined nucleotide) with 100 nM LigD_*Bs*_, in the presence of 100 nM of the indicated ribonucleotide. The figure is a composite image from different parts of the same experiment (see Supplementary Information). (**c**) Determination of the minimal number of nucleotides for intramolecular self-annealing. The assay was performed as described in Materials and Methods by incubating 5 nM of the indicated DNA with 100 nM LigD_*Bs*_ and 100 nM UTP. The dsDNA region of the hybrid DNA molecules is the same as in (**b**). The figure is a composite image made from different parts of the same experiment (see Supplementary Information).

The 45mer ligation product observed in these experiments could result from either the joining of two independent DNA molecules (end-joining), or from the folding-back of the overhanging 3’ terminus (snap-back structure depicted in Fig. 4a). To distinguish between these possibilities, LigD_*Bs*_ was simultaneously incubated with two different DNA substrates (only one of which was labeled, see schematics in Fig. 4b). These substrates both harbored a protruding 3’-end but differed exclusively in the nucleotide adjacent to the 5’-P end (dAMP and dGMP in the labeled and unlabeled substrates, respectively, in the left panel; reversed in the right panel). The unlabeled DNA was added at a 4:1 molar ratio (unlabeled:labeled; see schematics in Fig. 4b). As shown in the left panel, elongation and ligation occurred exclusively in the presence of UTP. The absence of products in the presence of CTP suggests that ligation did not occur between two DNA molecules (end-joining), but rather through snap-back in which the 3’-end folds back and uses the nucleotide adjacent to the 5’P end as a template. Consistently, when the base adjacent to the 5’-P group was dGMP (right panel), ligation products were only observed in the presence of CTP. The incubation of those substrates with LigD_*Bs*_ resulted in a 1-nucleotide extension of the labeled strand (27mer), which was subsequently ligated to the recessed 5’-P end. These results confirm that the incoming ribonucleotide is added to the 3’-end of the protruding strand.

Based on the results shown in Fig. 3 and 4, it could be concluded that the faster-migrating ligation product previously shown in Fig. 2a (lane *m*), is the result of intramolecular snap-back by pairing of the 3’-terminal dTMP in DNA-X and the dAMP present in the position adjacent to the orphan nucleotide. By the contrary, the presence of a 3’-terminal dAMP in DNA-B precluded the intramolecular ligation, the LigD_*Bs*_/Ku_*Bs*_ complex only rendering the AP incision product (Fig. 2a, lane *j*).

Having demonstrated that LigD_*Bs*_ can generate and further ligates intramolecular loops on these substrates, we next determined the minimum number of nucleotides required to form those loops. In previous assays, the 3’-protruding end was 8 nucleotides long. We therefore tested substrates with progressively shorter 3’-overhangs (8 to 4 nucleotides), all containing dAMP adjacent to the 5’-P terminus (Fig. 4c). In the presence of UTP, LigD_*Bs*_ produced ligation products of 45, 44, and 43 nucleotides when the 3’-overhang was 8, 7, and 6 nucleotides long, respectively. No ligation products were observed when the overhang was 5 nucleotides or shorter, indicating that a minimum of 6 nucleotides is required for LigD_*Bs*_ to successfully form the loop structure.

### The synapsis promoted by Ku_***Bs***_ prevents intramolecular loop formation during end-joining of complementary ends

As it can be observed in Fig. 2a (lane *m*), Ku_*Bs*_ does not prevent DNA-X from adopting the snap-back conformation. Therefore, the absence of this conformation when substrate A is present (lane *n*) suggests that pairing of the protruding 3’-end of substrate DNA-A with the complementary 3’ overhang of substrate X prevents the snap-back conformation. These findings indicate that when there is at least 5-nucleotide complementarity between the 3’ overhangs of the upstream and downstream DNA molecules, the synapsis promoted by Ku_*Bs*_ prevents the self-ligation of the DNA by LigD_*Bs*_.

**Fig. 5:**
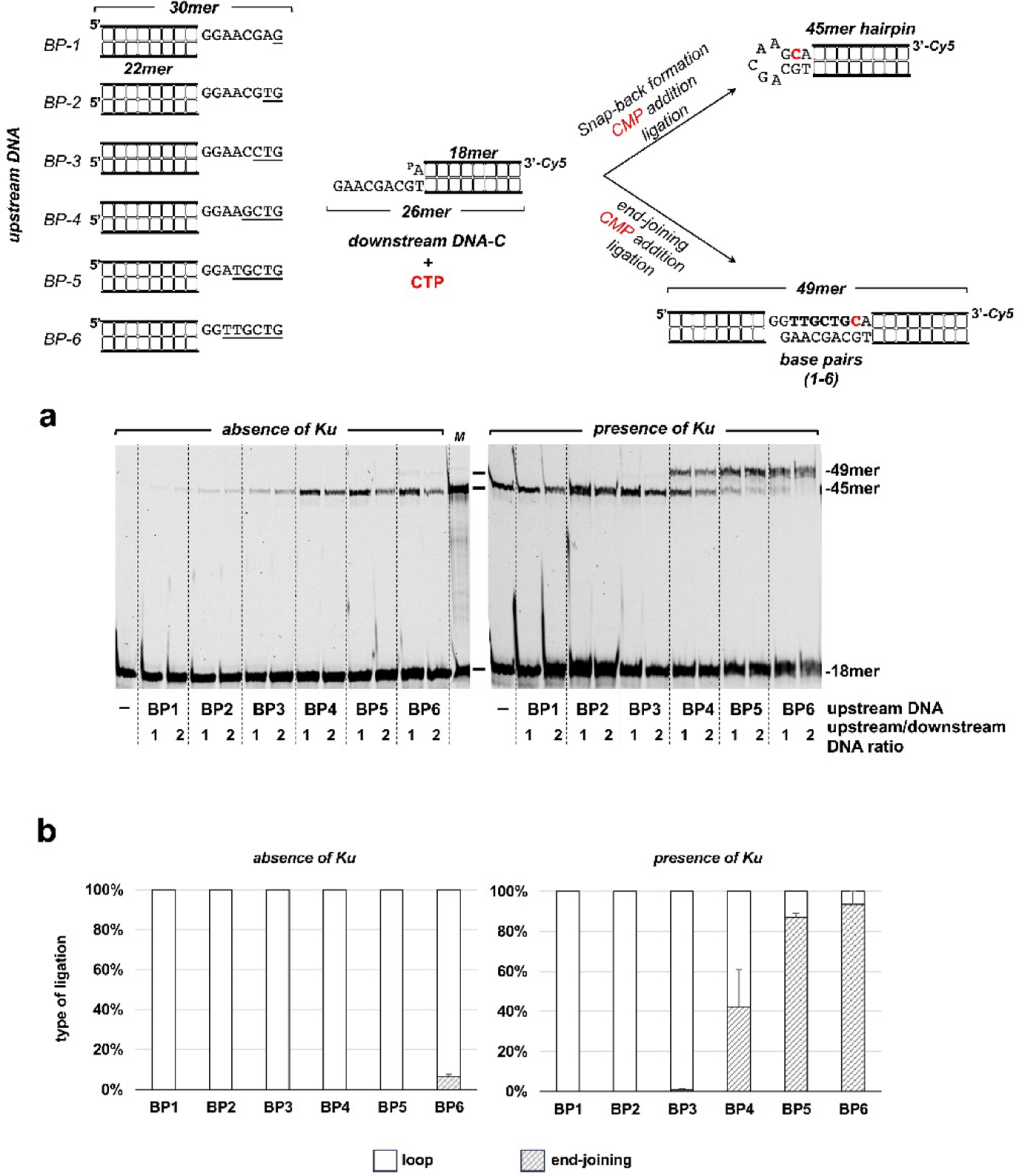
Determination of the minimum number of complementary bases between protruding 3’-ends required to prevent intramolecular DNA ligation. (**a**) The upper scheme shows the hybrid molecules used in the assay and the potential ligation products. The upstream DNAs have different number of 3’ overhang nucleotides complementary to the 3’ overhang nucleotides of the downstream molecule. The complementary bases between the upstream DNA (BP1-6) and the downstream DNA are underlined. The assay was performed as indicated in Materials and Methods, by incubating 20 nM of labeled downstream DNA with the specified upstream molecule at the indicated ratio, 1 µM CTP, and 100 nM LigD_*Bs*_, in the absence (left panel) or presence (right panel) of 100 nM Ku_*Bs*_. M: 45mer-Cy5 marker. The figure is a composite image made from different parts of the same experiment (see Supplementary Information). (**b**) Quantification of the 45mer and 49mer ligation products from three independent experiments (Mean ± SEM). Only the products corresponding to the upstream/downstream DNA ratio 2:1 were quantified.

To determine the minimum number of complementary bases for end-joining to become favored over snap-back formation, we performed analogous experiments using a series of upstream molecules (BPX, where X varied from 1 to 6; see schematics at the top of the Fig. 5). These molecules had varying degrees of complementarity to the 3’ overhang of the downstream molecule. As shown in Fig. 5a (left panel), when Ku_*Bs*_ was absent, LigD_*Bs*_ promoted hairpin (snap-back) formation regardless of the degree of complementarity between the 3’ overhangs. By contrast, when Ku_*Bs*_ was present (Fig. 5a, right panel), end-joining was favored when the upstream and downstream overhangs could form at least 4–5 base pairs (Fig. 5a, b).

### Role of Ku_***Bs***_ regions during the NHEJ reaction

**Fig. 6:**
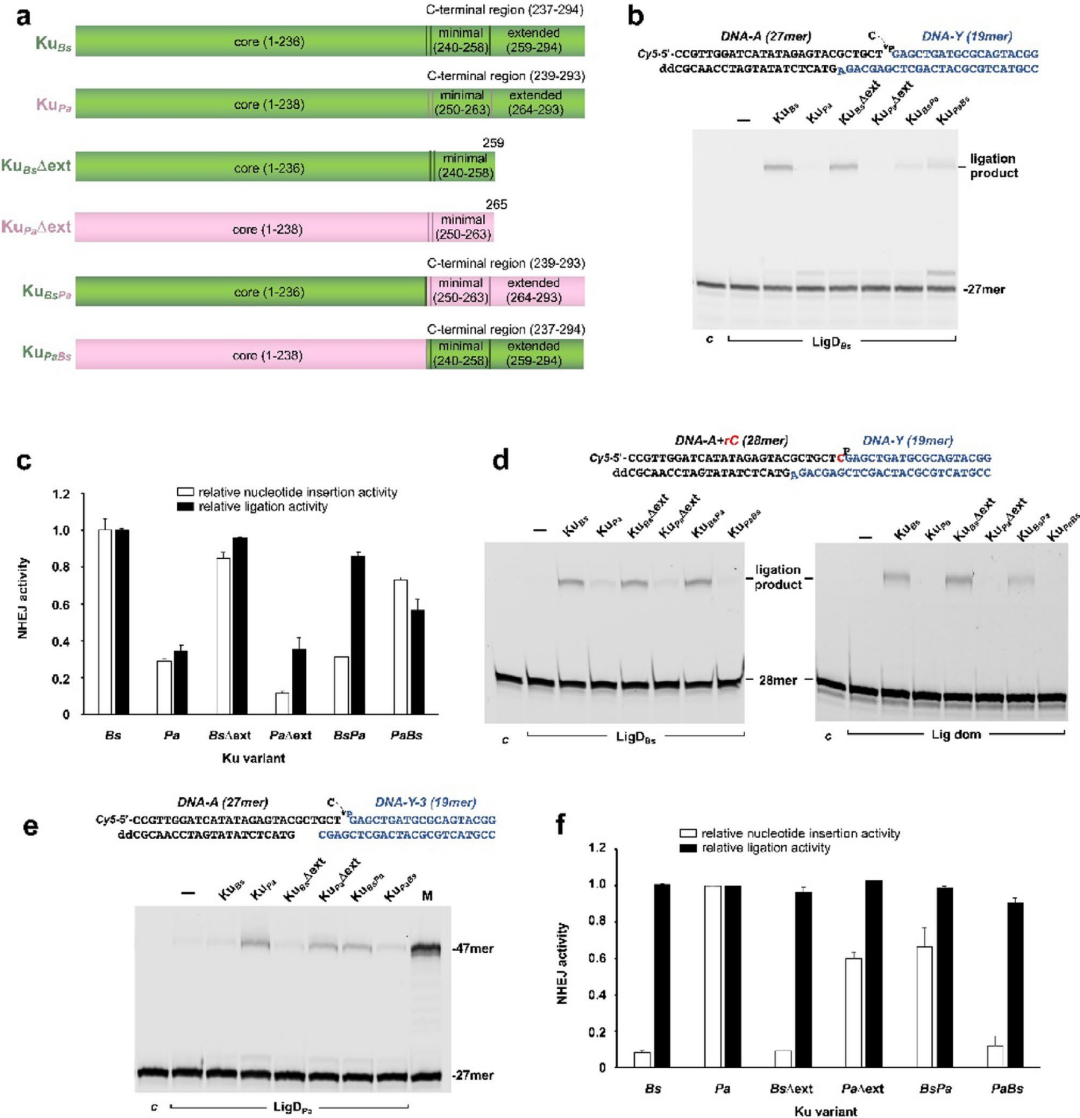
*Role of the Ku domains in the NHEJ reaction.* (**a**) Schematics of the Ku variants. (**b**) Effect of the Ku variants in the coupled nucleotide insertion + ligation reactions catalyzed by LigD_*Bs*_. The assay was performed as described in Materials and Methods by incubating 20 nM of the DNA substrates (depicted on top of the figure) with 100 nM LigD_*Bs*_, 50 nM of the indicated Ku variant, and 100 nM CTP. (**c**) Relative elongation and ligation products yielded by LigD_*Bs*_ in the presence of the Ku variants respect to the value obtained with Ku_*Bs*_ (1.0). Bar chart shows the relative nucleotide insertion activity [(28mer + ligation products)/total DNA], and the relative ligation activity [ligation products/(ligation + 28mer products] (*n* = 3; Mean ± SEM). (**d**) Effect of the Ku variants in the ligation reaction. Left panel, the assay was carried out as described in Materials and Methods by incubating 20 nM of the DNA substrates (depicted on top of the figure) with 100 nM LigD_*Bs*_, and 50 nM of the indicated Ku variant. Right panel, the assay was carried out as described in Materials and Methods by incubating 20 nM of the DNA substrates (depicted on top of the figure) with 20 nM of LigD_*Bs*_ ligase domain and 50 nM of the indicated Ku variant. (**e**) Effect of the Ku variants in the coupled nucleotide insertion + ligation reactions catalyzed by LigD_*Pa*_. The assay was carried out as described in Materials and Methods by incubating 20 nM of the DNA substrates (depicted on top of the figure) with 50 nM LigD_*Pa*_, 50 nM of the indicated Ku variant, and 100 nM CTP. M: Cy5-47mer marker. (**f**) Relative elongation and ligation products yielded by LigD_*Pa*_ in the presence of the Ku variants respect to the value obtained with Ku_*Pa*_ (1.0). Bar chart shows the relative nucleotide insertion activity [(28mer + ligation products)/total DNA], and the relative ligation activity [ligation products/(ligation + 28mer products] (*n* = 2; Mean ± SEM).

As described above, all prokaryotic Ku proteins share a conserved central *core* domain. This is followed by a C-terminal region comprising two distinct elements: a small minimal Cter domain and the electropositively charged ext Cter domain^12^. Incubation of LigD_*Bs*_ with truncated Ku_*Bs*_ variants, lacking either the ext Cter domain alone or both the minimal Cter and ext Cter domains, revealed the importance of the minimal Cter domain for the interaction with LigD_*Bs*_, and a role for the ext Cter in controlling DNA threading and bridging^12^. These previous studies on the influence of the Ku regions on LigD activity were limited to the sealing function of the enzyme. We extended this by examining how Ku regions influence the coordinated polymerization and ligation activities required for NHEJ. To study the role of the *core* and the C-terminal region, and to minimize potential artifacts associated with protein shortening, we generated Ku variants by exchanging these regions between Ku_*Bs*_ and Ku_*Pa*_ that have a similar length, 294 and 293 residues (according to^12^), respectively, thereby preserving the overall length of Ku. Thus, two chimeric proteins were made by swapping the C-terminal regions (minimal + extended Cter) between Ku_*Bs*_ and Ku_*Pa*_ resulting in Ku_*BsPa*_ (Ku_*Bs*_* core* + Ku_*Pa*_ C-terminal region) and Ku_*PaBs*_ (Ku_*Pa*_* core* + Ku_*Bs*_ C-terminal region) (Fig. 6a and Supplementary Fig. S2). Additionally, the truncated variants Ku_*Bs*_Δext and Ku_*Pa*_Δext were constructed by introducing stop codons at positions 260 and 266 of the genes coding for Ku_*Bs*_ and Ku_*Pa*_, respectively, resulting in proteins containing only the *core* + minimal Cter domains of Ku_*Bs*_ and Ku_*Pa*_ (see Materials and Methods).

We tested the ability of LigD_*Bs*_ to perform the nucleotide insertion and subsequent ligation reactions using those Ku variants and the two 3’ protruding DNA molecules depicted in Fig. 6b (note that only one of the molecules is 5’-labeled), whose synapsis would render a 1-nucleotide gapped molecule. In the absence of Ku, LigD_*Bs*_ failed to give rise to any product (Fig. 6b), confirming the requirement of Ku for NHEJ. As expected, LigD_*Bs*_ was very efficient in inserting CMP (28mer + ligation products) in the presence of Ku_*Bs*_ and Ku_*Bs*_Δext. The reduced amount of reaction products by LigD_*Bs*_ with Ku_*Pa*_ is consistent with the existence of specificity in the interaction between LigD and its cognate Ku. Whereas a similar reduction in the amount of insertion products was observed in the presence of Ku_*Pa*_Δext and Ku_*BsPa*_ (Ku_*Bs*_* core* + Ku_*Pa*_ C-terminal region), the CMP insertion activity was restored with Ku_*PaBs*_ (Ku_*Pa*_* core* + Ku_*Bs*_ C-terminal region), underscoring the critical role of the minimal Cter domain of Ku_*Bs*_ in mediating specific interaction with LigD_*Bs*_ during the initial nucleotide insertion step (see Fig. 6b). Quantitative analysis of the ligation efficiency [ligation products/(28mer + ligation products)] showed that the ligation step is largely independent of the C-terminal region of Ku (see Fig. 6c), as near wild-type ligation efficiency was observed with Ku_*Bs*_Δext and with Ku_*BsPa*_. Altogether, these results emphasize the importance of the Ku *core* during the ligation reaction. Consistently, when a similar experiment was performed using two DNA hybrids whose synapsis generates a nick that can be directly sealed without the need for a prior nucleotide insertion step (see scheme in Fig. 6d), LigD_*Bs*_ yielded a ligation product with Ku_*Bs*_, Ku_*Bs*_Δext and Ku_*BsPa*_ (Fig. 6d, left panel). A similar result was obtained when the assay was carried out using the independently expressed C-terminal LigDom of LigD_*Bs*_ (Fig. 6d, right panel), strongly suggesting a specific interaction between the Ku *core* and the ligase domain during the ligation step. When the experiments were performed with LigD_*Pa*_, the maximal end-joining activity occurred in the presence of Ku_*Pa*_, Ku_*Pa*_Δext and Ku_*BsPa*_, all containing the minimal Cter domain of Ku_*Pa*_, reinforcing the importance of this Ku region in the interaction with LigD (Fig. 6e). However, the relative percentage of ligation was similar in all cases (Fig. 6f), suggesting differences in the interaction between LigD_*Pa*_ and Ku_*Pa*_ during the NHEJ reaction.

## Discussion

Bacterial NHEJ relies on a Ku homodimer and the multifunctional ATP-dependent LigD^9,10^. Extensive *in vitro* and *in vivo* studies of these two proteins have provided a detailed understanding of how NHEJ operates in bacteria (reviewed in^20^). In brief, the pathway is initiated when Ku recognizes and binds the ends of a DSB by threading the DNA through its open ring, promotes the synapsis of both ends and recruits LigD that fills the gaps generated after synapsis and completes repair by ligating the ends.

**Fig. 7:**
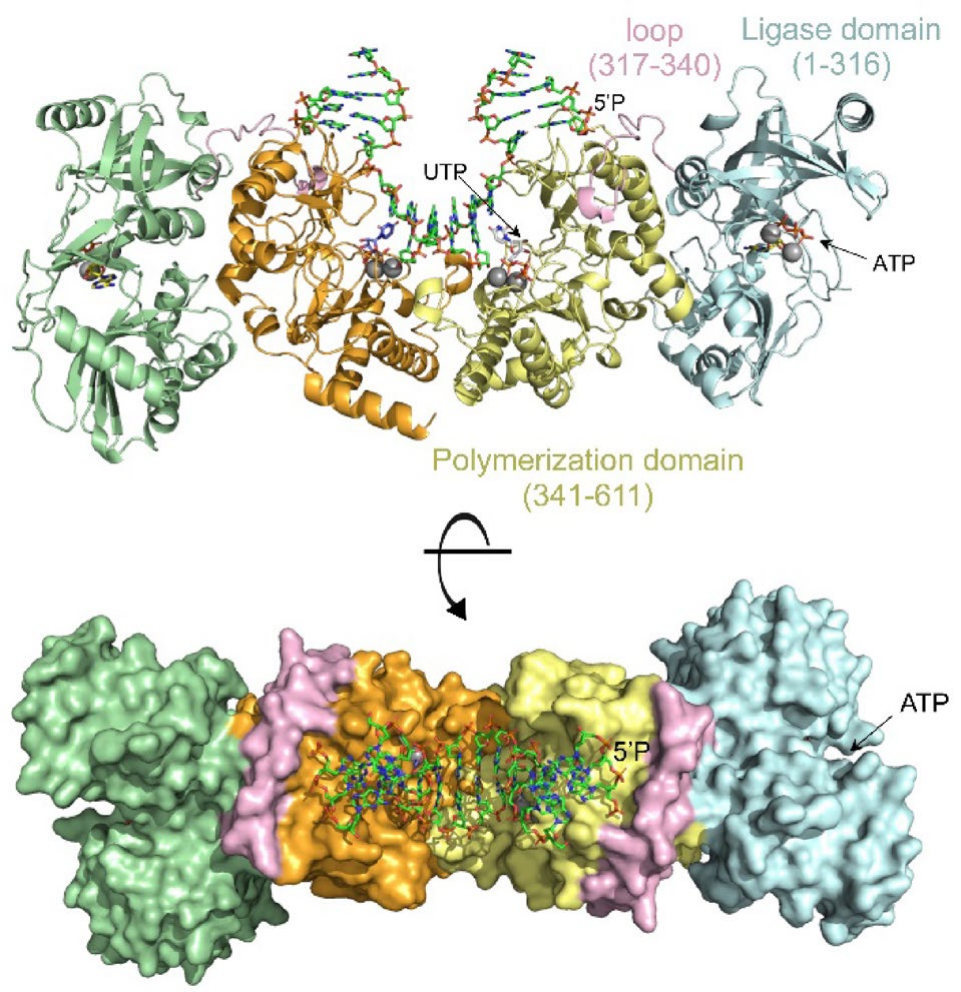
*Structural model of an annealed DSB bound to LigD*_*Bs*_*.* The figure was generated by superimposing the AlphaFold-predicted structural model of LigD_*Bs*_ onto the crystallographic structure of the *M. tuberculosis* PolDom bound to an annealed DSB [^35^; PDB 4MKY]. To determine the potential location of the two metal ions and the ATP molecule in the active site of the LigD_*Bs*_ LigDom (in blue), the crystallographic structure of the *M. tuberculosis* LigDom [^49^; PDB 6NHZ] was structurally fitted to the LigD_*Bs*_ model. The predicted structure of LigD_*Bs*_ was obtained through the Uniprot web site (https://www.uniprot.org/) from the Alphafold Database ID AF-O34398-F1^48^, and used under CC BY4.0. Figure was generated using The Open-Source Pymol MolecularGraphics System, v. 2.5.0, Schrödinger, LLC (Open-Source PyMOL is Copyright (C) Schrodinger, LLC.)

The *in vitro* biochemical results presented here using the *B. subtilis* NHEJ system show that LigD_*Bs*_ is able to execute all the catalytic steps during the NHEJ reaction without dissociating from the DNA (Figs. 1 and 2). The extensive structural studies of the *Mycobacterium tuberculosis* PolDom (*Mt*PolDom) in complex with a dsDNA bearing a recessed 5’P-terminus revealed the formation of a preternary-precatalytic complex^34^. This complex is stabilized by the *Mt*PolDom interaction with the recessed 5’P-end, the binding of two Mn^2+^ ions at the active site, and the presence of an incoming nucleotide complementary to the templating base adjacent to the 5’P-end^34^. This specific complex arrangement is proposed to facilitate the in *trans* extension of an incoming primer from the opposing end of the break. Further crystallographic structures of the *Mt*PolDom-DNA synaptic complexes with dsDNA containing 3’ protruding ends that are self-complementary demonstrated the formation of two *Mt*PolDom-DNA binary complexes oriented toward each other. In this configuration, the 3’ overhang functions simultaneously as the template strand for one *Mt*PolDom monomer and as the primer strand for the other^35^. Therefore, once PolDom has performed the in *trans* extension, it must dissociate to allow the LigDom to access the 5’P-end to catalyze the ligation reaction. Despite the absence of any full-length LigD-DNA synaptic complex structure, and considering that both domains recognize the same 5’P-end, it is plausible that their active sites are spatially separated to prevent competition that could inhibit the NHEJ reaction. The superimposition of the Alphafold predicted structure of the full-length LigD_*Bs*_ [AF-O34398-F1^36,37^] onto the crystallographic structure of the *Mt*PolDom NHEJ synaptic complex (PDB 4MKY,^35^) reveals that the active sites of the ligase and polymerization domains are oriented in opposite directions (see Fig. 7). This spatial rearrangement implies that during NHEJ reaction PolDom must first disengage from the resulting nick after nucleotide incorporation followed by repositioning of the LigDom to access and seal the nick. Therefore, our results indicate that the dynamic interaction of LigD with Ku is stable enough to support these sequential domain rearrangements, ensuring an efficient repair of the DSB.

To understand the dynamics of the Ku-LigD interaction, a series of studies were published in which the functional interaction of both proteins was analyzed using truncated versions of Ku lacking either the ext Cter or the ext Cter + minimal Cter domains^12,16,26^. A prior study showed that the minimal Cter domain of Ku_*Bs*_ is essential for recruiting LigD_*Bs*_, while the extended domain restricts Ku translocation along DNA ends^12^. Gel filtration assays assessing direct Ku_*Bs*_-LigD_*Bs*_ interactions indicated a weak, DNA-independent association between the two proteins in solution. Notably, the complete lack of interaction between LigD_*Bs*_ and the Ku_*Bs*_ variant containing only the *core* domain, along with the incapacity of this variant to enhance the ligation activity of LigD_*Bs*_ *in vitro*, led the authors to conclude that the Ku_*Bs*_* core* domain does not participate in LigD_*Bs*_ binding. More recent single-molecule forceps experiments showed that the central Ku_*Bs*_* core* domain is sufficient to bridge two complementary DNA ends, and that LigD_*Bs*_ increased the synaptic lifetime of the Ku_*Bs*_* core*^16^. Here, we have further broadened this investigation by assessing how the different regions of Ku_*Bs*_ modulate the coupling between the polymerization and ligation steps during NHEJ. The results presented here (Fig. 6b, c) support the importance of the initial interaction between LigD_*Bs*_ and the minimal Cter domain of Ku_*Bs*_, as previously described^12^, enabling the initial gap filling step. In this sense, Alphafold predictions of Ku_*Bs*_/LigD_*Bs*_ complex bound to DNA suggest that the α-helix that conforms the minimal Cter domain (residues 240–258) packs against the N-terminal ligase domain of LigD_*Bs*_, forming the primary interface between Ku_*Bs*_ and LigD_*Bs*_, allowing PolDom to form the preternary-precatalytic complex needed for the NHEJ reaction (see Supplementary Fig. S3). After nucleotide insertion, the final ligation depends on the interaction of LigD_*Bs*_ with the *core* domain of Ku_*Bs*_ (Fig. 6b, c), this latter finding supporting the previously proposed contribution of this domain in LigD_*Bs*_ interaction^16^. The results obtained with the *B. subtilis* system regarding the involvement of the Ku *core* domain in the interaction with LigD_*Bs*_ are analogous to those obtained with the *P. aeruginosa* NHEJ system, where the ligation activity of LigD is stimulated by a Ku variant containing the *core* domain alone (Fig. 6e, f and^11^). Likewise, it has been recently described that the ligase activity of *M. tuberculosis* LigD (LigD_*Mt*_) is also stimulated by both, the minimal Cter and the *core* of the cognate Ku^26^. In the same study, it was also shown that LigDom of LigD_*Mt*_ interacts with the *core* of Ku. The observation that both, the Ku_*BsPa*_ chimera and the Ku_*Bs*_Δext deletion mutant (each containing the core of Ku_*Bs*_) support ligation reactions of the LigDom of LigD_*Bs*_ as efficiently as the wild-type Ku_*Bs*_ (Fig. 6d) reinforces previous findings on the importance of the Ku *core* for LigD interaction^11,16,26^, and specifically at least with the ligase domain^26^.

Collectively, these results suggest distinct modes of interaction between Ku_*Bs*_ and LigD_*Bs*_ throughout the NHEJ reaction. Once Ku brings the two DNA ends together, LigD is recruited through a direct and specific interaction between the minimal Cter domain of Ku and, likely, the LigDom of LigD (supported by Alphafold models). In fact, such an interaction would prevent the catalytic center of the LigDom to interact with the 5’P terminus of the break, ensuring the correct formation of the precatalytic complex for the subsequent in *trans* nucleotide addition reaction, thereby preventing an NHEJ abortion. After the polymerization activity has filled any potential gaps, LigD transitions to interact with the Ku *core* region, probably also through the LigDom. This second engagement would allow the disengagement of the PolDom from the 5’-P end, permitting the 3’-OH and 5’-P ends to subsequently bind to the LigDom active site for the final sealing step.

As mentioned above, although the results obtained with LigD_*Pa*_, are also compatible with an interaction between LigD_*Pa*_ and the minimal C-ter domain during the initial gap filling reaction, the similar eficiency observed in the subsequent ligation with all the Ku variants (Fig. 6e, f) suggests differences in the interaction between LigD_*Pa*_ and Ku_*Pa*_ during the NHEJ reaction. In this context, it is worth noting that LigD_*Pa*_ contains an additional N-terminal PEDom that could contribute to those dissimilarities.

Our results also have revealed that LigD_*Bs*_ possesses an intrinsic ability to generate snap-back structures through the formation of intramolecular loops (Fig. 2–5), highlighting a structural flexibility that may influence the outcome of bacterial NHEJ reactions. Although this feature is not unique to LigD_*Bs*_, as MtPolDom has previously been shown to stabilize snap-back intermediates on substrates bearing 16-nucleotides 3’-overhangs^38^, LigD_*Bs*_ requires much less structure to act: only the 3’ end of at least 6-nucleotides long protruding terminus must be complementary either to the base adjacent to the 5’P end (for a direct ligation) or the preceding base (to fill the gap and seal the nick). These findings highlight the importance of preventing nucleolytic degradation of DNA ends resulting from breakage, not only to limit the potential loss of genetic information, but also to avoid the formation of self-ligated ends, which could have detrimental consequences for cell survival. In this context, Ku plays essential protective roles, as its binding to DSBs not only recruits LigD, but also protects DNA ends from nucleolytic degradation^12^, as described also for eukaryotic Ku^39–41^, and prevents their intramolecular ligation by promoting proper synapsis (Fig. 5). Interestingly, a similar challenge is found in eukaryotic Pol theta-mediated end joining (TMEJ), where DNA polymerase θ (Polθ) catalyzes repair of 5’-3’ resected DNA ends using short nucleotide microhomologies^42^. Sequencing of TMEJ repair products has shown that Polθ, like LigD_*Bs*_, can exploit internal short homologies to initiate snap-back synthesis^43,44^, thereby increasing the number of potential microhomology pairs available during subsequent stages of repair^43,45,46^. However, recent single molecule analyses indicate that snap-back structures generated during these events directly compete with productive synapsis^47^, raising mechanistic questions about how Polθ ultimately resolves extended snap-back intermediates during TMEJ. Therefore, despite the evolutionary distance between bacterial NHEJ and eukaryotic TMEJ, both systems must overcome similar structural limitations at DNA ends, relying in specialized proteins, Ku in bacteria and Polθ in eukaryotes, to prevent non-productive intramolecular configurations and ensure successful end joining.

## Conclusions

This study demonstrates that a single molecule of LigD_*Bs*_ is capable of performing all three potential catalytic steps of bacterial NHEJ: AP site incision, nucleotide insertion, and ligation in a fully processive manner without dissociating from the DNA substrate. This finding underscores the efficiency and coordination between LigD_*Bs*_ and Ku_*Bs*_ during the end-joining reaction. Furthermore, the role of Ku_*Bs*_ extends beyond DNA end protection and synapsis, as it plays a protective role by preventing the formation and further ligation of intramolecular DNA loops. To that, Ku_*Bs*_ favours the pairing of complementary protruding 3’-ends that would otherwise lead to aberrant self-ligation events that could compromise the accuracy and efficiency of the repair process. The use of chimeric and truncated Ku variants highlights the specificity of the Ku-LigD interaction and reveals distinct functional contributions of the Ku domains throughout the NHEJ process, modulating LigD_*Bs*_ activity during the DSB repair. Specifically, the minimal C-terminal domain of Ku_*Bs*_ is shown to be essential for initial recruitment of LigD_*Bs*_ to facilitate the nucleotide insertion step, while the Ku *core* domain contributes to the final ligation phase. These results collectively support a model for a dynamic interaction between LigD_*Bs*_ and Ku_*Bs*_ that orchestrates the efficient repair of DSB by a sequential handoff mechanism where LigD_*Bs*_ shifts from interacting with the C-terminal domain of Ku_*Bs*_ to the central Ku_*Bs*_* core* as the repair reaction progresses.

Although dynamic, LigD-Ku interaction has to be stable enough to prevent premature LigD dissociation before concluding the NHEJ reaction. Therefore, this work broadens our understanding of the bacterial NHEJ repair system, shedding light in the mechanistic interaction between Ku_*Bs*_ and LigD_*Bs*_ and provides a model of how bacterial cells could perform efficient and accurate repair of DSB under conditions where homologous recombination is not possible.

## Supplementary Information

Supplementary Information.

## Data Availability

The data underlying this article are available in the article and in its Supplementary Information. The predicted structure of LigD_*Bs*_ was obtained through the Uniprot web site (https://www.uniprot.org/) Alphafold ID AF-O34398-F1 [48] and used under the Creative Commons Attribution 4.0 International (CC BY 4.0) License. The dataset corresponding to experimental structures analyzed during the current study are available in the Protein Data Bank under the following accession numbers: crystallographic structure of the *M. tuberculosis* PolDom bound to an annealed DSB [4MKY](https:/www.wwpdb.org/pdb?id=pdb_00004mky) [35]; crystallographic structure of the *M. tuberculosis* LigDom [6NHZ](https:/www.wwpdb.org/pdb?id=pdb_00006nhz) [49]; crystallographic structure of *M. tuberculosis* Poldom preternary-precatalytic complex [3PKY](https:/www.wwpdb.org/pdb?id=pdb_00003pky) [34].
