# Supplementary material for: Insights into the functional coordination of LigD and Ku in bacterial nonhomologous end joining
Source: Sci Rep. 2026 Apr 9;16:16190. doi: 10.1038/s41598-026-47294-z (PMC13201618; doi:10.1038/s41598-026-47294-z)

Supplementary Information

Insights into the Functional Coordination of LigD and Ku in Bacterial Nonhomologous End Joining

Alicia del Prado, Amalia Buitrago, Adrián de Rus-Moreno, Iza O. Bienkowska, Ana de Ory, Silvia Díaz-Arco and Miguel de Vega*

Centro de Biología Molecular Severo Ochoa (Consejo Superior de Investigaciones Científicas-Universidad Autónoma de Madrid), Nicolás Cabrera 1, Madrid 28049, Spain.

*To whom correspondence should be addressed: mdevega@cbm.csic.es; Tel.: +34 911964717

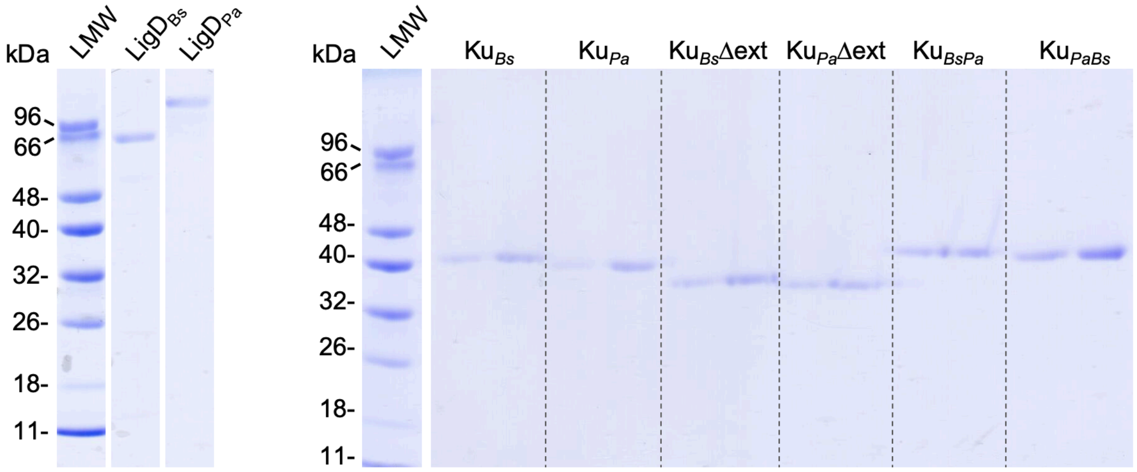

Figure S1. 12% SDS-PAGE gel of the purified proteins analyzed in this study. LMW: Low Molecular Weigth Protein Marker II from Nzytech

.

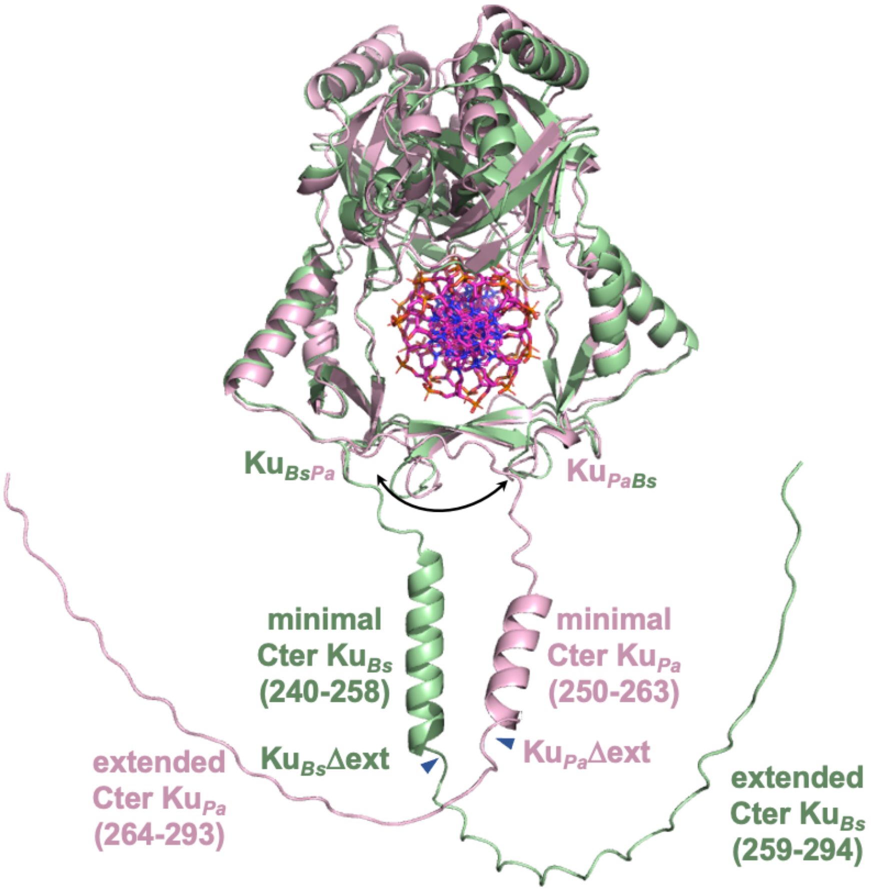

Figure S2. Superposition of the AlphaFold-predicted structural model of Ku_{Bs} (in green) and Ku_{Pa} (in pink) dimers. For simplicity, only a C-terminal domain of each dimer is represented. The curved double-headed arrow would point to the residues from which the extended C-terminal region of Ku_{Bs} and Ku_{Pa} have been swapped to get the Ku_{BsPa} and Ku_{PaBs} variants. The blue arrowheads indicate the position in Ku_{Bs} and Ku_{Pa} where the stop codon was inserted to get variants $Ku_{Bs}\Delta$ext, and $Ku_{Pa}\Delta$ext. Figure was generated using The Open-Source Pymol MolecularGraphics System, v. 2.5.0, Schrödinger, LLC (Open-Source PyMOL is Copyright (C) Schrodinger, LLC.)

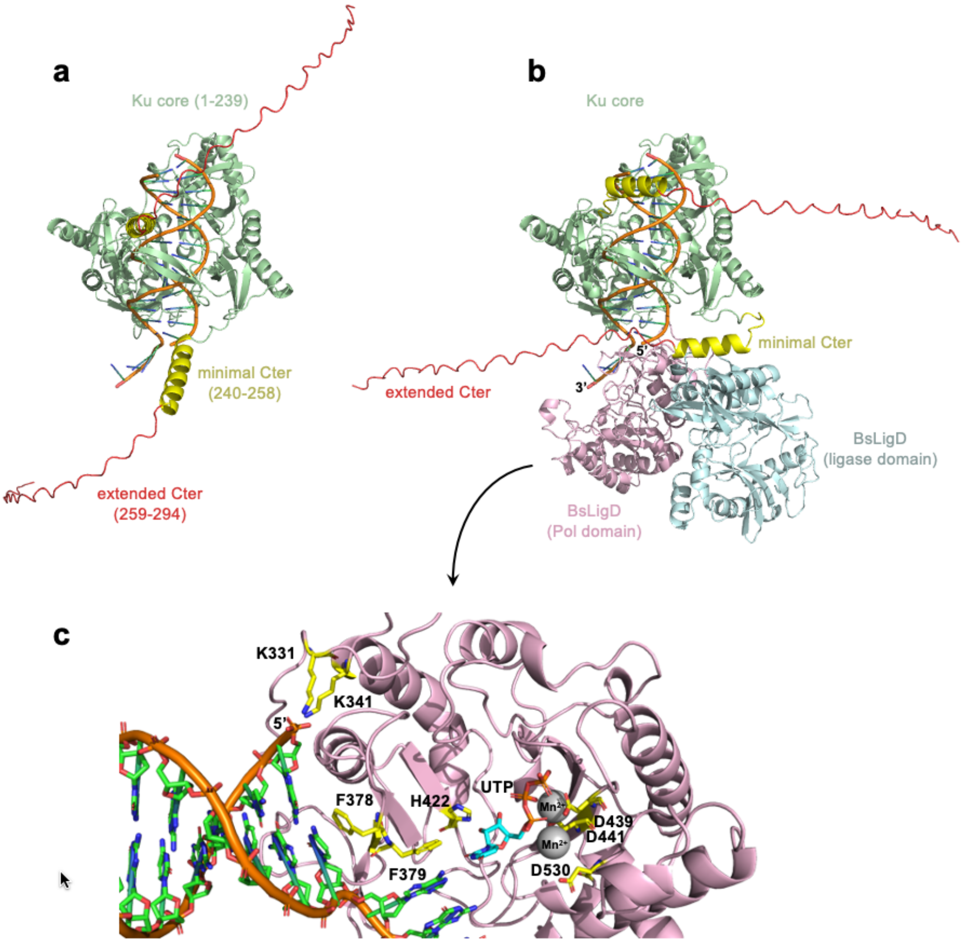

Figure S3. *Structural model of LigD$_{Bs}$/Ku$_{Bs}$ complex bound to DNA*. (**a**) Predicted structural model of Ku$_{Bs}$ dimer bound to a DNA containing a recessive 5'-end. Ku *core* domain, minimal Cter region, and extended Cter region are colored in green, yellow and red, respectively. DNA backbone is colored in orange. Structural model was generated by AlphaFold Server [1]. (**b**) Structural model of the complex LigD$_{Bs}$/Ku$_{Bs}$ dimer bound to a DNA containing a recessive 5'-end. Polymerization and ligase domains are colored in pink and cyan, respectively. Structural model was generated by AlphaFold Server [1] (**c**) Detailed view of the LigD$_{Bs}$ Pol domain bound to the dsDNA. Conserved residues that interact with DNA, as well as the aspartates that conform the catalytic triad are shown in yellow sticks. The incoming UTP and Mn^{2+} ions have been modeled by superimposing the crystallographic structure of *M. tuberculosis* Poldom preternary-precatalytic complex ([2]; PDB 3PKY) on the AlphaFold-predicted structural model of LigD$_{Bs}$/Ku$_{Bs}$ bound to DNA. Figure was generated using The Open-Source Pymol MolecularGraphics System, v. 2.5.0, Schrödinger, LLC (Open-Source PyMOL is Copyright (C) Schrodinger, LLC.)

1. Abramson, J. *et al.* Accurate structure prediction of biomolecular interactions with AlphaFold 3. *Nature* **630**, 493–500 (2024).

2. Brissett, N. C. *et al.* Structure of a preternary complex involving a prokaryotic NHEJ DNA polymerase. *Mol. Cell* **41**, 221–31 (2011).

Original uncropped gels

Figure 1: NHEJ of partially complementary DNA ends by LigD$_{Bs}$/Ku$_{Bs}$ under single DNA binding conditions

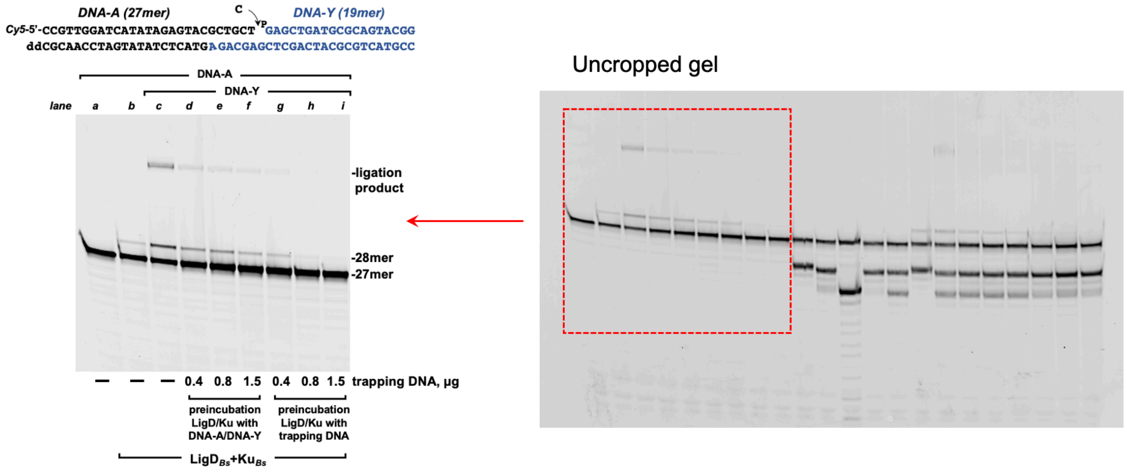

Figure 2 (a): NHEJ of partially complementary DNA ends containing a terminal AP site.

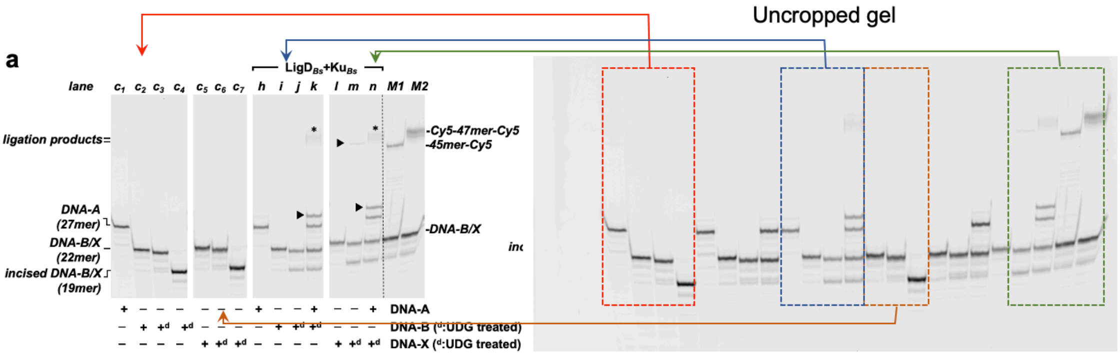

Figure 2 (b): NHEJ of partially complementary DNA ends containing a terminal AP site under single DNA binding conditions

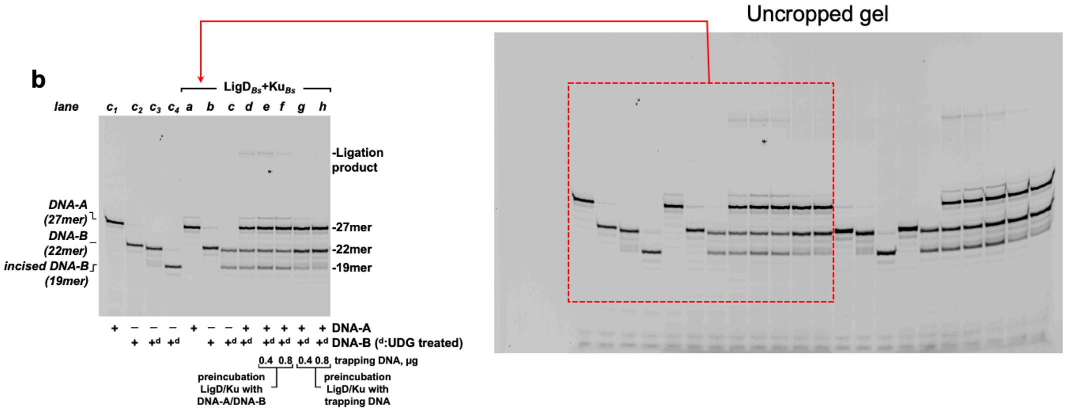

Figure 3 (**a**) Effect of the complementarity between the terminal base of the protruding 3'-end and the base preceding the orphan nucleotide in the formation of the ligation product.

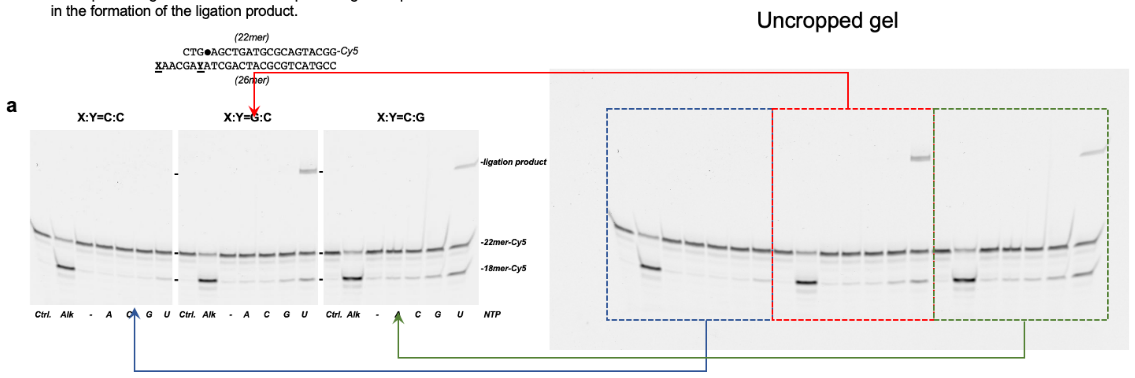

Figure 3 (**b**): Analysis of the complementarity between the incoming nucleotide and the base adjacent to the 5'P-end.

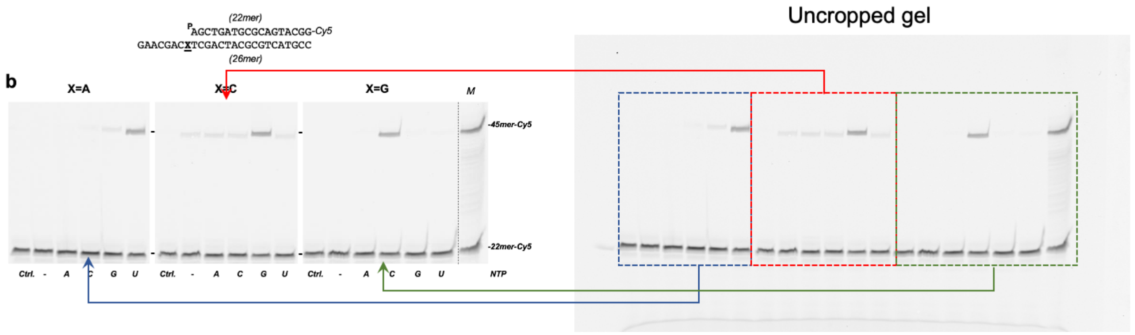

Figure 4 (**b**) Discrimination between the intra- and intermolecular models for 3' extension by $LigD_{Bs}$.

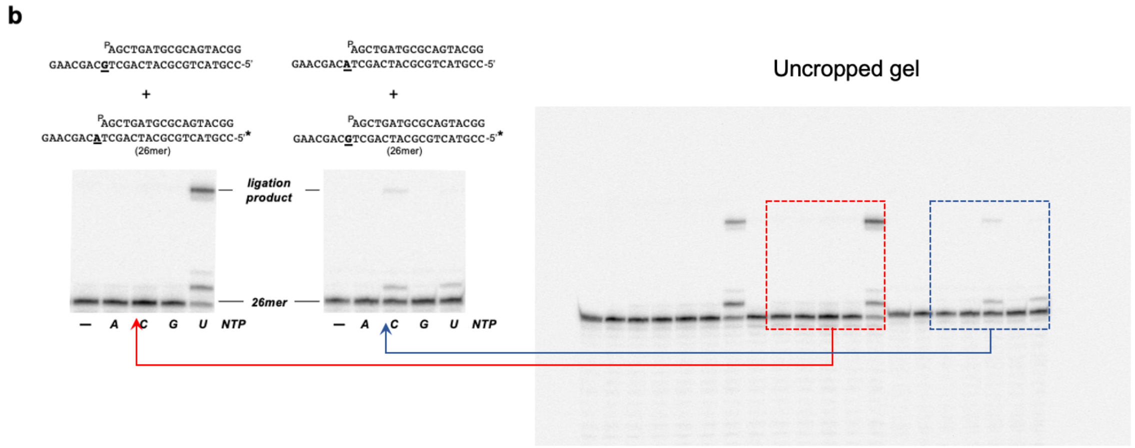

Figure 4 (**c**): Determination of the minimal number of nucleotides to allow intramolecular self-annealing

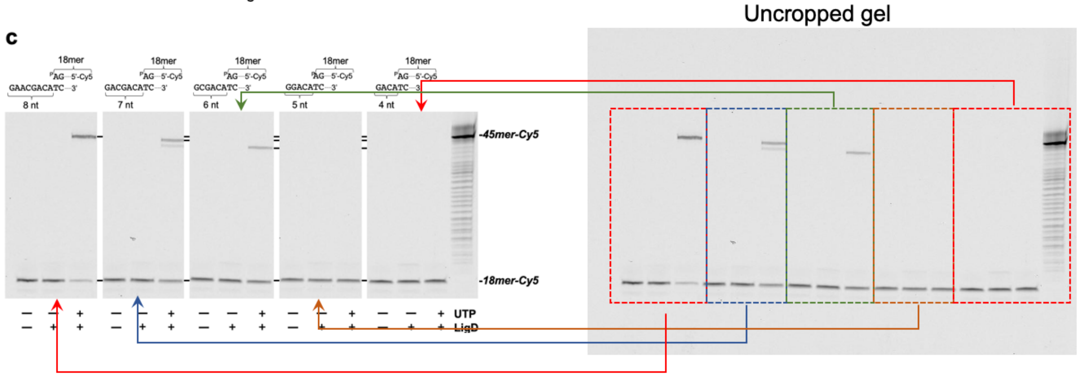

Figure 5 (**a**)

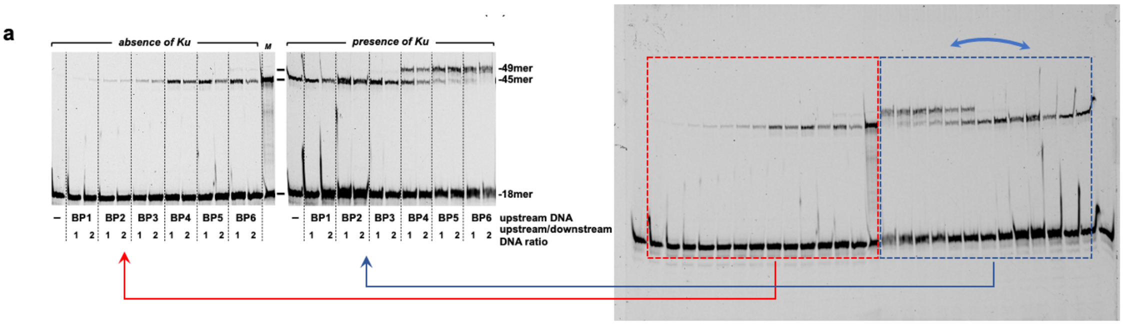

Figure 6 (**b**). Effect of the Ku variants in the coupled nucleotide insertion +ligation reactions catalyzed by $LigD_{Bs}$ during a NHEJ reaction

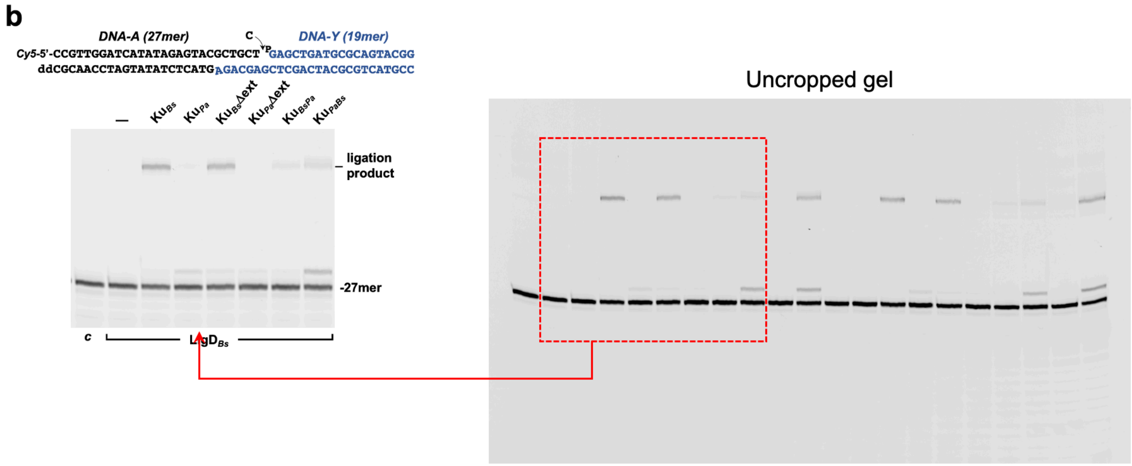

Figure 6 (**d, e**): Effect of the Ku variants in the ligation reaction during a NHEJ reaction

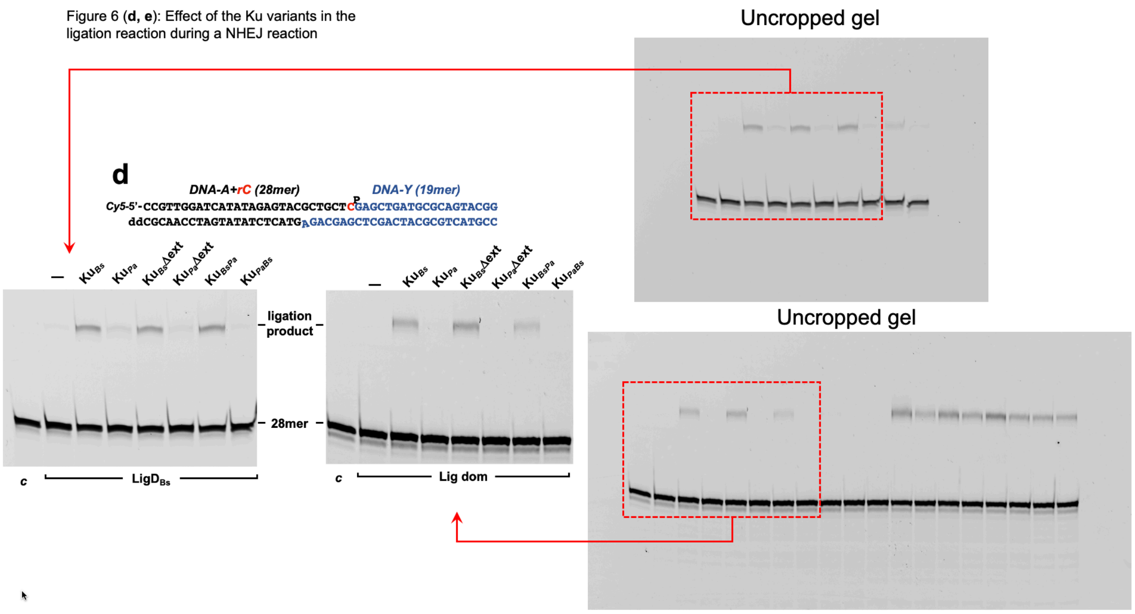

Figure 6 (**f**): Effect of the Ku variants in the ligation reaction during a NHEJ reaction

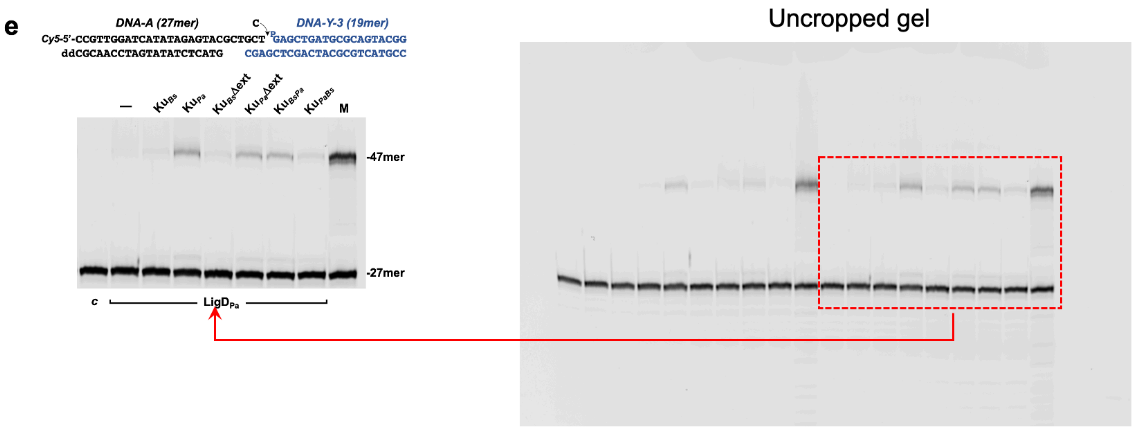

Figure S1. 12% SDS-PAGE gel of the purified proteins analyzed in this study.

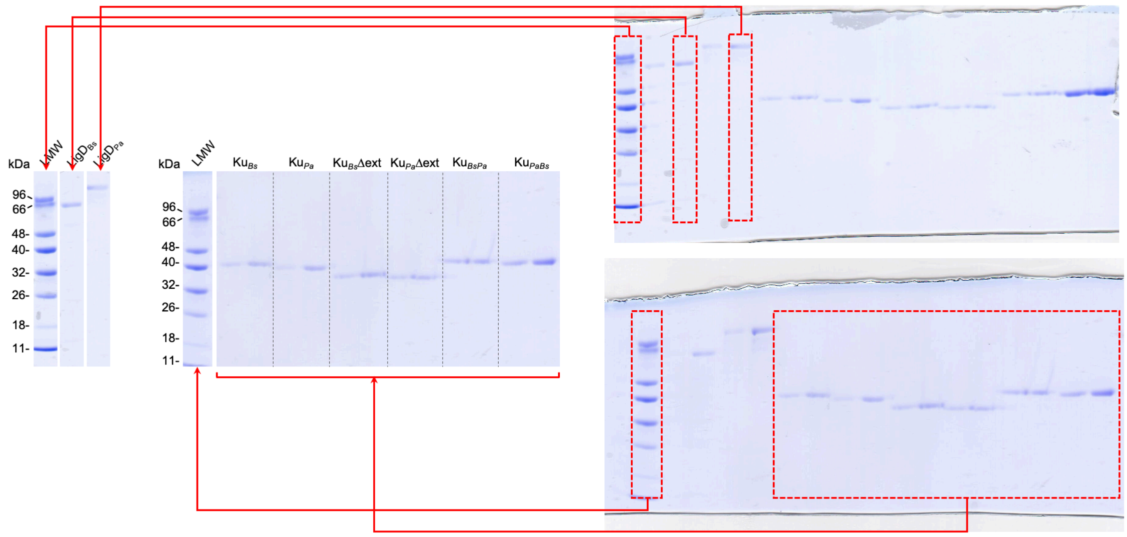

Supplement: Supplementary file 1 — Supplementary Information. [file 41598_2026_47294_MOESM1_ESM.pdf]
